# Elimination of Viroids from Tobacco Pollen Involves a Decrease in Propagation Rate and an Increase of the Degradation Processes

**DOI:** 10.3390/ijms21083029

**Published:** 2020-04-24

**Authors:** Jaroslav Matoušek, Lenka Steinbachová, Lenka Záveská Drábková, Tomáš Kocábek, David Potěšil, Ajay Kumar Mishra, David Honys, Gerhard Steger

**Affiliations:** 1 Biology Centre of the Czech Academy of Sciences, Department of Molecular Genetics, Institute of Plant Molecular Biology, Branišovská 31, 37005 České Budějovice, Czech Republic; jmat@umbr.cas.cz (J.M.); kocabek@umbr.cas.cz (T.K.); ajaymishra24@umbr.cas.cz (A.K.M.); 2 Institute of Experimental Botany of the Czech Academy of Sciences, Rozvojová 263, 165 02 Prague 6, Czech Republic; steinbachova@ueb.cas.cz (L.S.); l.zaveska.drabkova@ueb.cas.cz (L.Z.D.); david@ueb.cas.cz (D.H.); 3 Mendel Centre for Plant Genomics and Proteomics, Central European Institute of Technology, Masaryk University, Kamenice 5, 625 00 Brno, Czech Republic; david.potesil@ceitec.muni.cz; 4 Institut für Physikalische Biologie, Heinrich-Heine-Universität Düsseldorf, D-40204 Düsseldorf, Germany

**Keywords:** AFCVd and CBCVd propagation and eradication, viroid replication, viroid degradation, *Nicotiana tabacum*, male gametophyte, small RNA, recombinant AGO, TUDOR S-nuclease, strand-specific viroid RT-qPCR

## Abstract

Some viroids—single-stranded, non-coding, circular RNA parasites of plants—are not transmissible through pollen to seeds and to next generation. We analyzed the cause for the elimination of apple fruit crinkle viroid (AFCVd) and citrus bark cracking viroid (CBCVd) from male gametophyte cells of *Nicotiana tabacum* by RNA deep sequencing and molecular methods using infected and transformed tobacco pollen tissues at different developmental stages. AFCVd was not transferable from pollen to seeds in reciprocal pollinations, due to a complete viroid eradication during the last steps of pollen development and fertilization. In pollen, the viroid replication pathway proceeds with detectable replication intermediates, but is dramatically depressed in comparison to leaves. Specific and unspecific viroid degradation with some preference for (−) chains occurred in pollen, as detected by analysis of viroid-derived small RNAs, by quantification of viroid levels and by detection of viroid degradation products forming “comets” on Northern blots. The decrease of viroid levels during pollen development correlated with mRNA accumulation of several RNA-degrading factors, such as AGO5 nuclease, DICER-like and TUDOR S-like nuclease. In addition, the functional status of pollen, as a tissue with high ribosome content, could play a role during suppression of AFCVd replication involving transcription factors IIIA and ribosomal protein L5.

## 1. Introduction

The haploid male gametophytes of higher plants play a vital role in plant fertility and crop production through the generation and transport of the male gametes to ensure fertilization and seed set. Plant male germ cells differentiate from somatic cells in the adult, resulting in generation of pollen grain consisting of two cell types differing dramatically in their cell fates. In tricellular pollen (i.e., *Arabidopsis*), two sperm cells are fully engulfed in the cytoplasm of a larger vegetative cell. In plant species shedding bicellular pollen (i.e., tobacco and hop) [1], sperm cells are originated by the division of generative cells later in the growing pollen tube. Sperm cells provide the paternal genetic contribution to the zygote and the endosperm, respectively, in double fertilization. The delivery of sperm cells is fully controlled by the pollen vegetative nucleus, which has decondensed chromatin compared to the compact sperm cell [2] and does not contribute DNA to the fertilized zygote nor to the endosperm [3,4]. Pollen possesses efficient mechanisms for the elimination of “parasitic nucleic acids”, such as transposable elements in the male germline; however, these mechanisms are different from those described in animals where 25–29 nt piwi-interacting RNAs are employed. In *Arabidopsis* pollen, transposable elements are reactivated and transpose exclusively in the vegetative nucleus, whereas they remain inactive in sperm cells. Moreover, 21 nt small interfering RNAs (siRNA) derived from the activated gypsy-class retrotransposons in the vegetative nucleus target the silencing of the respective transposons in sperm cells [5] utilizing the AGO1–AGO2–DCL4 pathway [6]. This mechanism requires the regulated and directed transport of siRNAs and their accumulation in sperm cells that is facilitated by the close cytoplasmic connection of sperm cells to the vegetative nucleus within the male germ unit [7].

During evolution, pollen from some plant species interacted with another type of “parasitic nucleic acid”, a viroid. Viroids are single-stranded, circular RNA molecules that replicate autonomously in infected host plants. Their lengths are 239–401 nucleotides, depending on the viroid species. As non-coding RNA, viroids do not code for any protein but possess the biological activity and function of a minimal parasite replicating autonomously in the host cell and promoting specific disease of the host, including significant losses in the yield of some important crops. The viroid RNA does encode signals within its sequence and its thermodynamically stable or metastable structures that allows it to exploit the host machinery for its own replication, processing, transport and induction of pathogenesis (for reviews see, e.g., [8,9,10]). Viroids are transcribed in a rolling-circle mechanism either in nuclei (*Pospiviroidae* with 27 species) or in chloroplasts (*Avsunviroidae* with four species) of plant hosts. Members of family *Pospiviroidae* possess a rod-like secondary structure in their thermodynamically optimal state, are localized primarily in the nucleolus and are replicated in an asymmetric rolling-circle mechanism by DNA-dependent RNA polymerase II (Pol II) of the host [11]. Only (+)-stranded replication intermediates are processed by host enzymes to monomeric circles. Viroids as non-coding “parasitic” RNAs [12,13] show fast evolutionary changes during the adaptation to new hosts, and their spreading is intensively facilitated by the agrotechnique (predominantly mechanical transmission) [12].

Some viroids are transmissible through plant generative phase, pollen and seeds [14,15,16,17,18,19,20,21,22,23]. Transmission routes via seeds and pollen are the major ways of infection propagation from parental plants to their progeny and distribution among individuals. The term “seed transmission” or vertical transmission means the passage of the pathogen through seeds to seedlings and plants in the next generation, while in horizontal transmission the pathogen is transmitted to other individuals via pollination with infected pollen (for review see, e.g., [24]). The degree of vertical and/or horizontal transmissions of viroids depends on plant species and cultivar; the viroid species and even certain point mutations in a viroid; and the infection stages and environmental conditions (see, e.g., [24,25]). For instance, pollen and seed transmissibility was detected for chrysanthemum stunt viroid and hop stunt viroid (HSVd) in infected tomato [15], avocado sun blotch viroid [26] and coconut cadang-cadang viroid [18]. Although no direct evidence has been provided, pollen transmissibility can be expected for some seed-transmissible viroids listed by Mink [17], such as citrus exocortis viroid (CEVd) in tomato and *Coleus blumei* viroid in *Coleus* [17,27]. Wan Chow Wah and Symons [19] confirmed the transmission of grapevine yellow speckle viroid 1 and HSVd via seeds in *Vitis vinifera*.

Some viroid species are lowly transmissible or non-transmissible by pollen. In general, the efficiency of vertical transmissibility is highly variable [17]. For instance, in the case of the well detectable potato spindle tuber viroid (PSTVd) in mature potato pollen, transmission through true seeds ranged from 66% to 35% [28]. Recently, Matsushita and Tsuda [21] described seed transmissibility of PSTVd in petunia; however, the authors were only able to detect this viroid by a sensitive RT-PCR-based method and not by in situ hybridization, suggesting low levels of PSTVd in petunia pollen. Importantly, some viroids were not detected in mature pollen of infected plants. For instance, apple skar skin (ASSVd) and dapple apple viroid (DAVd) are seed-transmissible, but were not detectable in apple pollen [16]. HSVd is pollen and seed-transmissible in tomato, but was non-transmissible through hop seeds [17] or true seeds of *Galinsoga cilliata*, one of the HSVd-susceptible weed plants contaminating hop fields [29]. CEVd is seed-transmissible in tomato but was not detectable in citrus seedlings derived from infected plants [30]. Consequently, some viroids can pass neither by vertical nor horizontal transmissions in corresponding host combinations and conditions, although they can be detected in floral organs or in somatic anther tissues because there is not anything blocking their trafficking to floral somatic tissues. This situation is obviously mediated by mechanism(s) that eliminate viroids or depress, significantly, their levels during pollen maturation or germination. As a consequence, viroid infection can be detected in such infected pollen only by very sensitive methods, or it is not detectable at all. Hop latent viroid (HLVd) elimination was detected during different developmental stages of hop pollen maturation [31], suggesting intimate molecular interaction(s) of the viroid parasitic RNA and its recognition and elimination on the level of the male gametophyte.

In the present work, we mainly concentrate to apple fruit crinkle viroid (AFCVd, 368–372 nts), which is a new hop viroid and of increasingly high theoretical and economical significance. We studied the elimination of this parasitic viroid RNA from the male germline of *Nicotiana tabacum* as a pollen model. AFCVd is a member of the family *Pospiviroidae*, genus *Apscaviroid* [32]. For some comparative analyses, we used another important viroid hop isolate: citrus bark cracking viroid (CBCVd, 283–286 nts), which is a member of genus *Cocadviroid*. Both viroids were preliminary tested and found to be non-transmissible via seeds on *Nicotiana benthamiana* as a common host [33]. Using specific tobacco transgenotes supporting viroid propagation and by the use of the combination of electrophoretic, hybridization, PCR-based and next-generation sequencing (NGS)-based methods, we show in this study that elimination mechanisms of viroids from tobacco pollen involve strong depression in their replication rates and viroid degradation.

## 2. Results

### 2.1. Infectivity of AFCVd and CBCVd in N. tabacum

**Figure 1:**
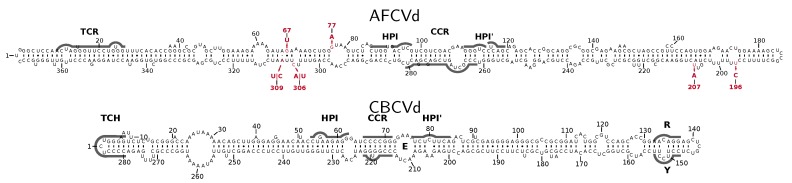
Secondary structures of analyzed viroids AFCVd and CBCVd. Sequences of AFCVd (AC AB104533) and CBCVd (AC KM211547) are shown with consensus structures predicted for alignments [35] of all available AFCVd and CBCVd sequences, respectively, optimized in ConStruct [36] at 37 °C, and drawn with R2R [37]. The central conserved region (CCR), extra-stable hairpin I (HPI), terminal conserved region (TCR), terminal conserved hairpin (TCH), loop E (E) and RY motif are marked. Variable nucleotides of AFCVd as detected in vd-sRNA (Figure S6) and by screening of cDNA libraries from germinating pollen tubes are given in red.

**Figure 2:**
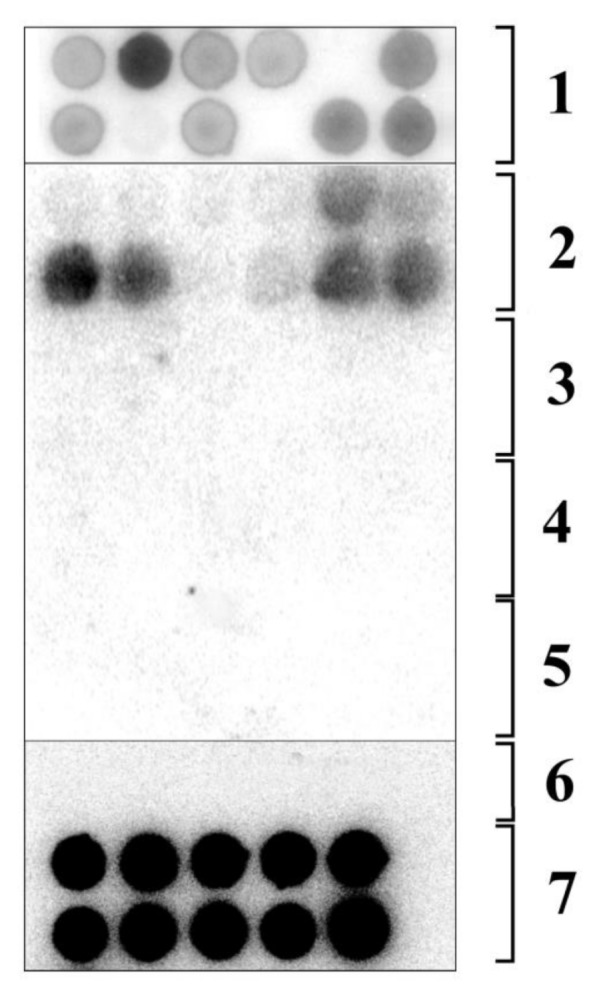
Dot-blot hybridization analysis of AFCVd-inoculated tobaccos and seedlings of F1 generation obtained by reciprocal crossings of AFCVd-infected plants and healthy controls. The dot blot was performed using oligoprime-labeled viroid cDNAs (see Materials and Methods for further details). Row 1, results of biolistic inoculation of *N. tabacum* with AFCVd RNA partly-purified from infected *N. benthamiana* (Table 1, row 3), eight weeks post inoculation (p. i.); row 2, AFCVd signals from *N. benthamiana* inoculated with raw RNA extract using Carborundum method, four weeks p. i.; row 3, 45 day old plants from seeds obtained by crossing of AFCVd-infected parental plants; row 4, 45 day old plants from seeds obtained by crossing uninfected control plants with AFCVd-infected parental plants; row 5, 45 day old plants from seeds obtained by crossing of AFCVd-infected parental plants with uninfected control plants; row 6, healthy controls; row 7, AFCVd signal in leaves of tobacco transformed with a plant vector containing infectious dimeric AFCVd driven from p35S. In rows 1–6, six samples, and in row 7, only five samples were spotted to one row.

We previously studied some physiological effects of AFCVd and CBCVd (Figure 1) in multiple infected hops [33]. These pathogens are not transmissible through seeds in hop plants (*Humulus lupulus*) or *N. benthamiana* (unpublished results). In the present work we aimed to analyze possible transmission or elimination of these viroids in *N. tabacum*, a species related to *N. benthamiana*. *N. tabacum* is known as a plant model for analysis of pollen physiology especially because it is possible to define developmental pollen stages according to lengths of buds [34]. To analyze whether tobacco can be host for these two pathogens, two inoculation methods were tested to achieve infection (Table 1). CBCVd infection of tobacco was not successful using Carborundum or biolistic methods with viroid DNA (full genomic restriction fragments or vector sequences; not shown) or RNA as inocula. AFCVd RNA, however, efficiently infected *N. tabacum* after biolistic inoculation. According to dot-blot hybridization (Figure 2), 75% of biolistically inoculated plants became infected (Table 1), suggesting that tobacco can be used as an experimental host for AFCVd and to analyze potential viroid elimination or transmissibility through pollen in this experimental model species.

### 2.2. Viroid Elimination from *N. tabacum* Pollen

Using reciprocal crossings, AFCVd was not transmissible through seeds, suggesting that there is no vertical transmission of this viroid via pollen; there was not any via female germline either (Figure 2). Pollination with pollen from infected plants did not infect a mother plant (not shown) via growing pollen tubes, demonstrating that it is also not transmissible by horizontal transmission. This suggests a total elimination of AFCVd from pollen or germinating pollen.

**Figure 3:**
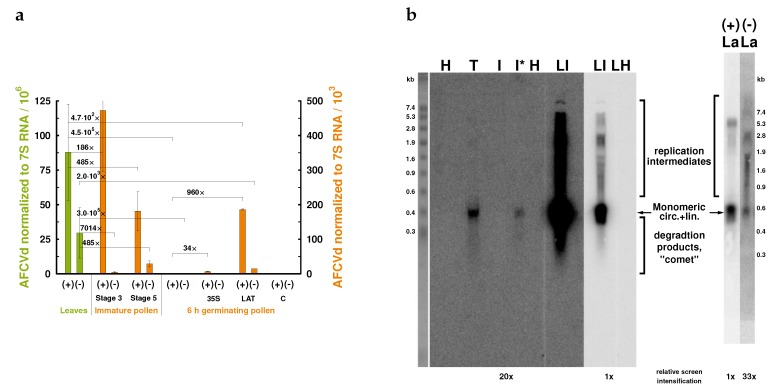
Levels of AFCVd in developing, mature and germinating pollen. (**a**) Strand-specific RT-qPCR profiles of AFCVd infection in different *N. tabacum* tissues. Total RNA was isolated from infected leaves and pollen at different stages, reverse transcribed for (+) or (−) AFCVd strands and their relative levels were assayed using qPCR and normalized to 7SL RNA. (+), (−), AFCVd sequences of (+) and (−) polarity, respectively; 35S, transformed pollen with 35S:AFCVd vector; LAT, plant transformed with AFCVd dimer under pLAT52 promoter; C, healthy control. (**b**) Northern blot analysis of AFCVd intermediates. Analysis was performed using 35 μg of total RNA per lane isolated from AFCVd infected *N. tabacum* tissues collected within the interval 90–120 dpi. Samples were probed either by [^32^P-dCTP]-labeled viroid cDNA or by [32P-dUTP]-labeled strand-specific RNA probes in lanes designated (+) and (−) with 1 × 10^6^ cpm per 1 mL of hybridization solution and hybridized as described in Materials and Methods. Membranes were scanned after 24 h exposure, relative screen intensification is given at the bottom of the figure. The marker ladder on the left is the ethidium bromide-stained RNA III marker (Boehringer Mannheim). The position of AFCVd monomer is indicated by an arrow. H and T, RNA from healthy and transformed 6 h-grown pollen tubes, respectively; I, RNA from infected 6 h-grown pollen tubes; I*, RNA from infected 6 h-grown pollen tubes after sample concentration by precipitation of LiCl-soluble RNA; LI, RNA from infected tobacco upper leaves; LH, RNA from healthy tobacco upper leaves; La, AFCVd in mature pollen transformed with plant vector pLAT56:bZIP18:AFCVd as detected by strand-specific probes.

We used qPCR and Northern blot hybridization to determine levels of viroid RNA in developing, mature-dormant and germinating pollen (Figure 3). While viroid was quite detectable in leaves, anthers and developing pollen during stage 3, it was hardly detectable at stage 5 and practically not detectable in mature pollen and growing pollen tubes by Northern blot analysis using total RNA extracts (Figure 3b). The radioactivity signal corresponding to AFCVd monomers was only detectable in infected pollen tubes if concentrated 2 M-LiCl-soluble RNA was used. This suggests that there are only some traces of viroid in growing pollen tubes (Figure 3b). Both (+) and (−) strands of AFCVd were unambiguously detectable by strand-specific RT-qPCR in extracts from infected, growing pollen tubes, although these levels were 4.5–3 × 10^−5^ times lower than in infected tobacco leaves (Figure 3a). The viroid traces represented about 5 fg of viroid per mg of pollen tissues (fresh mass) according to dilutions of viroid cDNA as qPCR standard. Altogether, these results suggest that pollen tubes still contained traces of viroid; therefore, this stage is a critical checkpoint for the elimination processes and for erasing of viroid from the male germline.

To understand the elimination processes in developing and, in particular, in mature pollen, levels of viroid were enhanced using plant transformation with two types of plant vectors containing dimeric (+) AFCVd driven with either 35S [33] or LAT52 promoters (see Figure S1 for corresponding plant vectors). The CaMV 35S promoter is known as strong for leaf and other somatic plant tissues, while LAT52 is a specific and rather strong promoter for late stages of pollen development and mature pollen. Indeed, the 35S promoter was rather weak in mature pollen, reaching about 16% expression in comparison to leaves, as observed with GFP mRNA as a marker for qPCR quantification. Using the 35S promoter, the level of AFCVd increased about 34-fold in growing pollen (Figure 3a). This level is, however, still dramatically lower than the viroid level in leaves. The pLAT52 vector with bZIP18 mRNA as a marker showed activity to a level close to actin mRNA in transgenic pollen (not shown). Dimeric AFCVd was inserted into the *Kpn*I restriction site of pLAT52 in a position between the 3’ end of bZIP18 and the polyA signal. Therefore, pLAT52:bZIP18:AFCVd (shortly named pLAT52:AFCVd) vector produced presumably non-polyadenylated and unstable bZIP18/AFCVd chimeras, because these were not detectable using AFCVd quantification by strand-specific RT-qPCR (see Materials and Methods), suggesting a quick degradation of non-polyadenylated bZIP18 after viroid processing. Only 3% of bZIP18 mRNA was detected using random priming in comparison to a polyadenylated variant. However, this expression mediated about 960-fold higher level of AFCVd in germinating pollen tubes (Figure 3a). Simultaneously, in transgenic pLAT52:AFCVd samples the characteristic longer-than-unit-length viroid intermediates were clearly visible on Northern blots (Figure 3b). Despite this significant increase in AFCVd levels, it was still about thousand times lower than the levels in infected (Figure 3a) or transformed leaves, which show high viroid accumulation to similar levels. The clear detection of (−) intermediates in pLAT52:AFCVd samples (Figure 3) suggests that true viroid replication proceeds in germinating pollen, but that the viroid replication pathway is under strong depression in pollen according to the dramatic viroid accumulation differences between leaves and pollen tissues.

Tobacco transformation with an infectious vector bearing dimeric CBCVd under control of 35S promoter (shortly 35S:CBCVd; Figure S1b) enabled us to express CBCVd in tobacco and to use tobacco infected with this viroid for some complementary analyses. In leaves, high levels of CBCVd were detected in transformed/infected plants and characteristic replication intermediates were detectable on Northern blots, suggesting intensive replication via rolling-circle replication. Much lower levels of this viroid were, however, detected in pollen and at stage 5 (Figure S2). Similarly to AFCVd, also for this viroid, only traces were detectable in the mature and germinating pollen, provided a high screen intensification (Figure S2). Traces of CBCVd replication intermediates were seen in gels for samples from mature and germinating pollen, suggesting that in this case viroid replication proceeds in pollen via the rolling-circle replication pathway.

### 2.3. Low Viroid Levels in Pollen did not Cause Strong PATHOGENIC Symptoms Connected to Pollen Vitality

**Figure 4:**
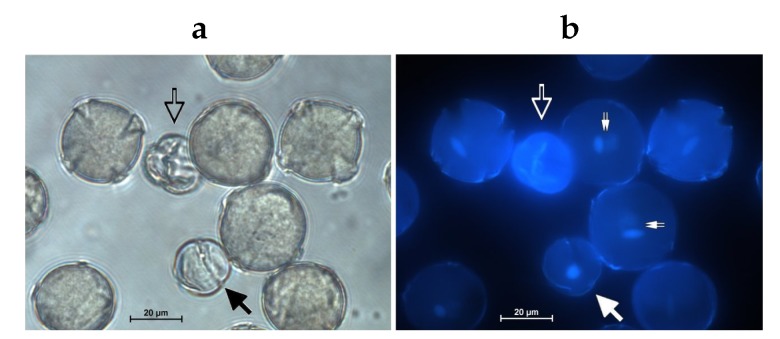
Microscopic analysis of individual pollen phenotypes evaluated in *N. tabacum* mature pollen grains. Aborted pollen is indicated in bright field (**a**) and fluorescence images after DAPI-staining (**b**) by empty arrows. Young bicellular pollen is indicated by filled arrows. Mature and late bicellular pollen grains with vegetative and generative nuclei visible under UV-light after fluorescence-DAPI staining are indicated on panel (**b**) by doubled arrows.

We checked the specific tobacco transgenotes supporting expression of AFCVd and CBCVd for some pathogenic symptoms on the pollen. AFCVd levels in pollen, even when enhanced using pLAT52:AFCVd transformation, did not induce significant pathogenic effects on pollen germination and vitality (Table 2, Figure 4): the level of aborted pollen was practically the same as in the control; the growth of pollen tubes showed some weak, but statistically insignificant depression; the pollen length distribution was not significantly different from healthy control. The same was true for transgenic variants expressing AFCVd under 35S promoter. These results suggest that some other process than a strong pathogenic reaction is responsible for viroid elimination.

### 2.4. Viroid Degradation in Pollen

**Figure 5:**
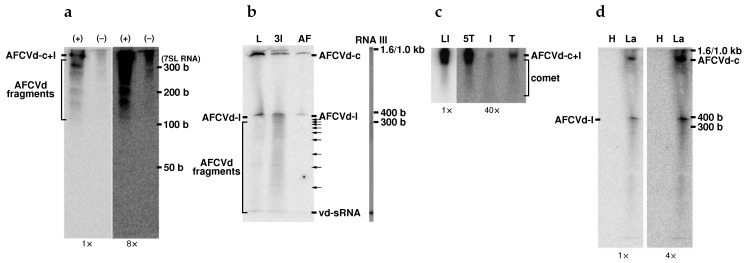
Figure 5. Northern blot analyses of shorter-than-unit-length degradation products of AFCVd in developing and germinating tobacco pollen: (**a**) 10 μg of 2 M-LiCl-soluble RNA isolated from AFCVd-infected pollen at developmental stage 3 was analyzed in 12% acrylamide gel containing 3 M urea and hybridized to strand-specific AFCVd RNA probes for detection of either (+) or (−) AFCVd sequences, as designated on top of the gel. (**b**) Analysis of 10 μg of 2 M-LiCl-soluble RNA isolated from AFCVd-infected tobacco leaves (L) and pollen from stage 3 (3I) using discontinuous-pH acrylamide gel electrophoresis. Samples were applied in 8% denaturing gel containing 8 M urea, hybridized with (−) AFCVd RNA probe for detection of (+) chains, monomeric circular (AFCVd-c) and linear (AFCVd-l) viroid. The arrows on the right indicate stronger degradation fragments of AFCVd from pollen stage 3. AF, partly purified AFCVd isolated using PEG fractionation of LiCl-soluble RNA. (**c**) Analysis of “comets” from tobacco infected leaves and pollen infected with AFCVd on 1.5% agarose gel; viroid-specific RNA was detected using hybridization to [^32^P]dCTP-labeled AFCVd cDNA. LI, 10 μg total RNA from infected leaves; 5T, I and T, 30 μg LiCl-soluble RNA from 35S:AFCVd vector-transformed pollen stage 5, infected germinating pollen and 35S:AFCVd vector-transformed germinating pollen, respectively. (**d**) Analysis of LiCl-soluble RNA from healthy (H) and vector pLAT52:AFCVd-transformed germinating pollen under similar gel conditions as in (**b**). After electrophoresis, nucleic acids in gels (**a**,**b**,**d**) were electroblotted to positively charged nylon membranes; a capillary blot was performed on Biodine A nylon membrane for gel (**c**). Positions of RNA markers are indicated on the right sides of the gels. Gel strip with RNA III marker was silver stained. As marker of 300 base RNA, position of 7SL RNA is indicated in (**a**). Positions of “comets” zones and cones where viroid RNA fragmentation is seen are indicated in this figure. Relative screen intensifications are given on the bottom of gels.

Northern blot analyses of both viroids (Figure 3b, Figure 5 and Figure S2) demonstrated an accumulation of shorter-than-unit-length degradation products hybridizing to viroid cDNA and forming some “comets” in agarose gels. These comets are much more intense for samples from developing, mature and germinating pollen than in samples from leaves, despite that the viroid replication in leaves proceeds obviously with much higher rate than in pollen. While the radioactivity signal corresponding to comet in leaf samples (Figure 5c) was about 10% of the total, the comet signal comprised from 50% to 60% in growing pollen tube samples.

Analysis of comet composition in acrylamide gels showed a predominant fraction of shorter-than-unit-length (100–300 nucleotides) but longer than 21–24-meric viroid-derived small RNA (vd-sRNA) for sequences of both (+) and (−) viroid polarity (Figure 5a). The analyses of the viroid degradation products from pollen developmental stage 3 on denaturing acrylamide gels in discontinuous pH gradient showed that both linear and circular viroid forms were detectable (Figure 5b). The ratio of circular to linear viroid, however, was very low (0.177) for pollen samples, while for leaves this ratio reached 2.2, suggesting a high degradation rate of circular AFCVd in pollen. The quite abundant, discrete fragmentation corresponding to shorter-than-unit-length viroid detected in the pollen samples in comparison to leaf samples (Figure 5b) suggests degradation rather than intensive blocking of processing and ligation at this developmental step.

We analyzed transgenic pollen with AFCVd under a LAT52 promoter to check for circular AFCVd in pollen tubes. Circular AFCVd was detectable in transgenic pollen tubes (Figure 5d) expressing about 960-fold more viroid than in infected pollen (Figure 3a). That is, despite the extremely low level of AFCVd, processing and ligation occurred also in growing pollen tubes, and a circular viroid has to be relatively resistant to quick degradation in pollen tubes.

We used strand-specific viroid RT-qPCR to quantify the ratios of (+) and (−) AFCVd strands in leaves, and mature-dormant and germinating pollen tubes, where critical viroid levels were detected (Figures S3 and S4), using extracts of total RNA with or without additional precipitation of long oligomeric replication intermediates with 2 M LiCl. In total RNA extracts, long replication intermediates were present, while in 2 M LiCl-soluble RNA long molecules were missing due to their precipitation (Figure S3). In 2 M LiCl-soluble extracts from mature and germinating pollen the ratios were similar, reaching 2.5× and 2.9× lower values in germinating and mature-dormant pollen, respectively (Figure S3c,d). In total RNA containing replication oligomers (i.e., without LiCl precipitation), similar levels of (+) viroid in mature-dormant and germinating pollen were present, while the levels of (−) chains were 8.2× lower in germinating versus mature pollen (Figure S3a,b), suggesting a significant shift to lower levels of (−) chains in pollen tubes.

These observations for AFCVd were confirmed by analysis of CBCVd, which shows a characteristic abundance of (−) chains in leaves of some plant species, including *Solanaceae* such as *N. benthamiana* and tomato [38]. Strand-specific viroid RT-qPCR was used to determine the ratio between levels of (−) and (+) strands for CBCVd in 35S:CBCVd transformed/infected pollen. The (−)/(+) ratio reached 5.7 for transformed/infected tobacco leaves (Figure S4), while it was “reversed” in pollen with values ranging from 0.38 in mature dormant pollen to 0.59 at developmental stage 5. These results suggest—in accordance to the observation with AFCVd—some shift to lower levels of (−) chains in pollen than in leaves, possibly due to a preferential degradation of (−) viroid strands.

### 2.5. AFCVd-Specific Small RNAs in N. tabacum

Next, we concentrated to small RNAs characteristically produced by the silencing machinery. To analyze composition and distribution of vd-sRNA, we used NGS to sequence small RNAs isolated from developmental stages 3 and 5 of AFCVd-infected developing pollen, as well as from growing pollen tubes of LAT52:AFCVd transgenic plants (Table S3). We used LAT52:AFCVd transgenic pollen tubes to analyze composition of small RNAs because the viroid level in infected germinating pollen is extremely low.

**Figure 6:**
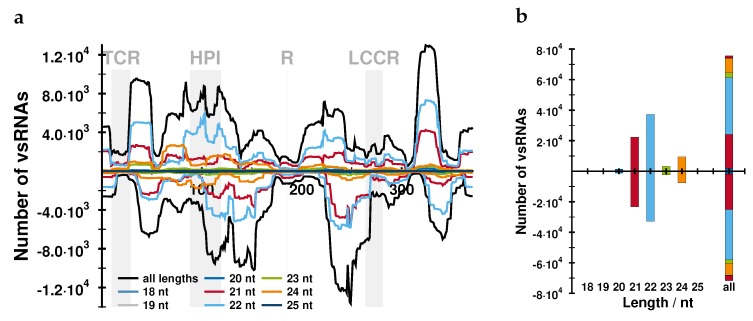
Mapping of vd-sRNA to the AFCVd genome (**a**) and length distribution of vd-sRNA (**b**) in infected leaves. (**a**) The x-axis represents the AFCVd genome; the curves above the x-axis (positive numbers) represent the number of vd-sRNAs with (+) sequence, below (negative numbers) with (−) sequence. The sequence regions of TCR, hairpin I, right terminal hairpin and lower CCR are marked (cf. Figure 1). Mapping of small RNAs was performed with bowtie v1.1.2 [39] allowing for one mismatch. For comparison to data from pollen stages 3 and 5 and pollen tubes, see Figure S5.

The distributions of vd-sRNA of either (+) or (−) polarity along the AFCVd genome were quite similar in leaves and in developmental pollen stages 3 and 5 from infected plants (Figure 6 and Figure S5a–e, and in leaves and pollen tubes from transgenic plants (Figure S5f–i). Differences in the “hotspots” of vd-sRNA production are due to mutations in the AFCVd progeny (see below): these mutations appeared spontaneously during AFCVd replication, even in the LAT52:AFCVd transgenic plants (Figure S6). The frequency of cleavage of the (+) and (−) chains at stages 3 and 5 of infected pollen seems to be similar, despite much lower levels of (−) AFCVd chains over (+) chains, suggesting that viroid chains of (−) polarity could be the critical point for the replication process.

In total eight base changes were identified in comparison to the original sequence in infected tobacco leaves and pollen stages by NGS of small RNAs. The differences included the following mutations: 67G→U; 77G→A; 196U→C; 207U→A; 306C→A; 306C→U; 309A→U and 309A→C (Figure 1 and Figure S6, Table S4). Most of these mutations appeared independently, i.e., during independent infections, suggesting some importance of these changes in the AFCVd offspring in tobacco. AFCVd cDNAs were cloned from infected leaves and from germinating infected pollen using concentrated 2 M-LiCl-soluble RNA extracts as sources of viroid RNA. In these viroid plasmid-libraries, six out of the eight mutations detected by NGS were also identified. Two mutations (67G→U and 306C→A) were not detected in the library from germinating pollen, where viroid traces were present, suggesting low level or full disappearance of these variants during the elimination process. Three out of nine mutated clones from germinating pollen were found to be truncated. Two of the mutants contained two mutations [196U→C, 207U→A] and [196U→C, 309A→U], respectively. The remaining four clones represented two triple mutated variants ([196U→C, 207U→A, 309A→C]; [196U→C, 207U→A, 309A→U]). The restricted extent of mutations contradicts a decreased fidelity of replication in pollen in comparison to leaves. Double and triple mutations suggest some successive adaptation of AFCVd to tobacco. Whether these AFCVd variants exhibited improved fitness in tobacco remains to be determined.

Vd-sRNAs of 21, 23 and 24 nt in length were present in leaves as well as in all pollen stages with frequencies f(22nt)>f(21nt)>f(24nt) (Figure 6 and Figure S5). DCL2 and DCL3, producing preferentially 22 and 24 nt sRNAs, respectively, are crucial in host defence against PSTVd and suppress viroid infectivity, while DCL4, producing preferentially 21 nt sRNAs, has a positive effect on viroid infectivity [40]. Analyses of 5’ ends of vd-sRNAs in pollen showed differences between leaves and developmental stages 3 and 5 in infected pollen, where many more vd-sRNAs with 5’C nts were detectable in pollen than in leaves (Figure S7). Specific accumulation of 5’C-ended vd-sRNAs at these developmental stages could possibly indicate a preferential involvement of *Nt*AGO5 in viroid attenuation via RISC complex in infected pollen [41,42]. The analysis of vd-sRNAs in pollen tubes transformed with LAT52:AFCVd vector showed several differences to the other developmental states:

1. The number of (−) reads in transformed/infected samples is lower than in the non-transformed samples (compare panels f with a or h with b in Figure S5). Note that the transgene produces (+) AFCVd while (−) strands have to stem from replication.
2. Mutations are also present in transgenic pollen tubes; these have to be due to ongoing replication. Their positions, however, are more restricted (196U→C mostly in (+); 207U→A, 309A→C, and 309A→U in similar amounts in (+) and (−)) than in the corresponding leaves (at least 67G→U and 77G→A in addition to those in pollen tubes).
3. Vd-sRNAs with 5’C are less preferred in transformed/infected pollen tubes than in infected pollen.

### 2.6. Levels of Possible Factors Mediating the Degradation Processes of Viroids

DCL factors and argonaute 5 (AGO5) homologues are candidates for mediating the degradation process of viroids in pollen according to the differences in vd-sRNAs observed in leaves and pollen. Furthermore, nucleases are clearly involved in this elimination process. According to some preliminary proteomics data (not shown), the Tudor S-like nuclease (TSN) was selected for analyses of its level in different developmental stages by qPCR.

**Figure 7:**
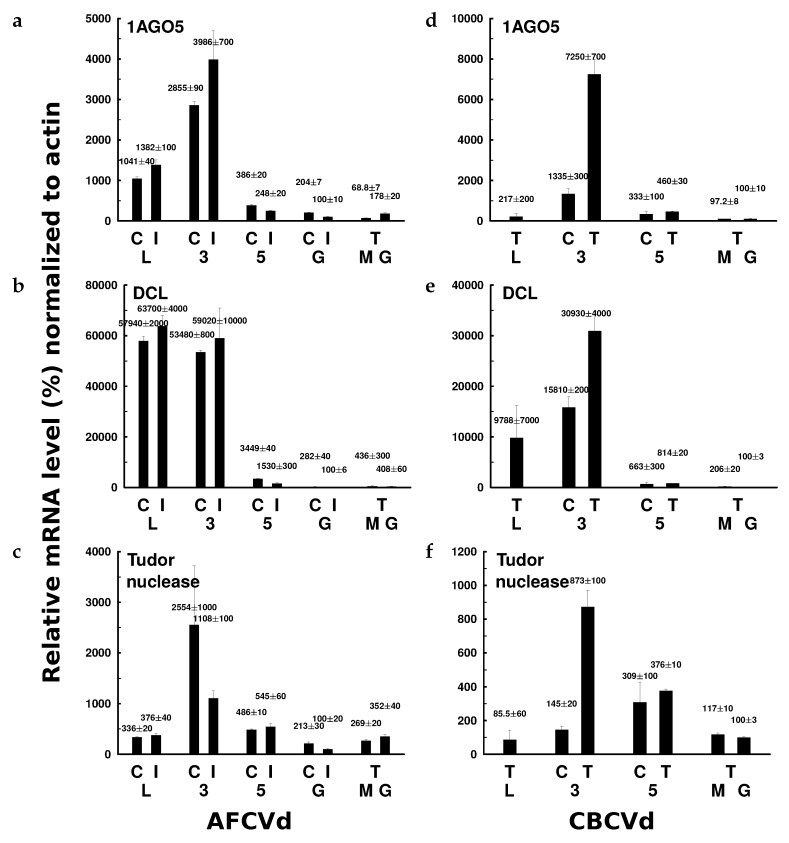
Relative mRNA levels of factors that could participate in viroid degradation in pollen: *Nt*AGO5 (**a**,**d**), *Nt*DCL (**b**,**e**) and *Nt*Tudor S-like nuclease (**c**,**f**). Result of qPCR performed as described in Materials and Methods were normalized to actin. Left: samples from AFCVd-infected or transformed plants; C and I, healthy control and infected sample, respectively; T, pLAT58:AFCVd-transformed/infected samples; L, young leaves; 3 and 5, pollen at developmental stages 3 and 5, respectively; G, germinating pollen (pollen tubes); M, mature pollen. Right: samples of 35S:CBCVd-transformed/infected plants; C and T, Lat52-transformed control and CBCVd-transformed/infected samples, respectively; L, young leaves; 3 and 5, pollen at developmental stage 3 and 5, respectively; M and G, mature CBCVd-transformed pollen and CBCVd-transformed pollen tubes, respectively.

The level of AGO5 mRNA in pollen stage 3 significantly increased in comparison to leaves for both AFCVd and CBCBd (Figure 7a,d). A strong decrease of AGO5 mRNA levels was observed in stage 5; the mRNA, however, was unambiguously detectable in low levels still in mature dormant pollen and in growing pollen tubes, suggesting availability of this factor for possible “cleaning” of viroid traces also during the pollen germination step. Similar profiles were seen for DCL molecules (Figure 7b,e): the highest levels were detected in early developmental stages followed by a strong decrease in later stages. In all cases a higher relative expression was seen in infected variants. The TSN profiles were somewhat different (Figure 7c,f): high levels were observed for stage 3 but TSN mRNA was also detected in dormant and germinating pollen, especially in CBCVd-transgenic samples. These results show the presence of selected factors that could participate in degradation of viroid to vd-sRNAs and/or to bigger fragments, which is in agreement with the observation of viroid levels (Figure 3) as well as with comets and degradation products seen in agarose and acrylamide gels (Figure 5).

AGO5 from *N. tabacum* was cloned (Figure S8a) and expressed in leaf sectors of *N. benthamiana* plants pre-infected for 28 days with CBCVd using infiltration with *A. tumefaciens* bearing a 35S:CBCVd construct at early, three-leaf stage. After expression of AGO5 or empty vector for 6 days in the right or left leaf sectors having similar position on the leaf blades, respectively, RNA was extracted and levels of (−) or (+) viroid strands were analyzed by qPCR. We used duplex strand-specific RT-qPCR; that is, RNA treatment, RT and PCR steps were performed in parallel for viroid and 7SL RNA reference gene. Statistically significant differences of (−)/(+) ratios of CBCVd chains in agroinfiltrated *N. benthamiana* were detected with and without *Nt*AGO5. This ratio changed from 1.42 in AGO-expressing sectors to 2.29 in controls, where a higher prevalence of (−) intermediates over (+) strands was observed, as is characteristic for CBCVd extracts from *N. benthamiana* (Figure S10b). This suggests some difference in viroid propagation influenced by this AGO, although by itself, *Nt*AGO5 could not induce strong CBCVd depression of propagation in *N. benthamiana* leaves. The same was true for the levels of AFCVd, which was not strongly attenuated in infiltrated *N. benthamiana* leaves (not shown).

### 2.7. The Model of Viroid Elimination Involving the Regulation of Translation Machinery in Pollen

Pollen represents a specific gene expression system, where transition of sporophytic to gametophytic developmental programs proceeds during its development, including significant changes to the level of transcription, such as downregulation of transcripts encoding Pol II and several associated proteins [43] and preparation of translational machinery in later stages of pollen development. The translational machinery is then activated from dormancy upon pollination to initiate fast proteosynthesis during the first steps of pollen germination and pollen tube growth. Proteosynthesis machinery includes regulation of several processes connected to 5S rRNA biogenesis by the ribosomal protein L5 (RPL5) and transcription factor IIIA (TFIIIA). Both these factors are able to bind PSTVd RNA [44]. According to some recent models [45,46] viroid propagation can be optimized via alternative splicing of TFIIIA to generate TFIIIA-7ZF, the process in which RPL5 acts as negative regulator; the TFIIIA-7ZF variant of TFIIIA is assumed to participate in the viroid replication cycle. Because viroid replication is under some depression in pollen, we analyzed TFIIIA and RPL5 mRNA levels during development of tobacco pollen in connection to the process of viroid elimination.

A relatively high level of TFIIIA was detected in healthy and infected tobacco leaves, lower relative mRNA levels of this factor were then quantified in healthy and infected pollen stage 3 and very low levels were detectable in pollen at late stages, mature and germinating pollen (Figure S11a). A similar profile was observed for CBCVd transgenic/infected tobacco, suggesting that TFIIIA is in principle not more abundant in developing pollen than in the leaves. The mRNA of RPL5 was quite abundant in developing pollen stage 3, and reached even higher level than in leaves (Figure S11c,d). These results are in good accordance with the assumption that these two factors could play some role in low rate of viroid propagation in pollen; that is, TFIIIA transcription is less available to generate a high portion of TFIIIA-7ZF variant, and, in addition, abundant RPL5 could mediate blocking of alternative TFIIIA transcription and accumulation of TFIIIA-7ZF.

In the attempt to demonstrate a possible role of TFIIIA-7ZF in AFCVd propagation, we cloned the homologue of this alternative TF from *N. benthamiana* infected with PSTVd AS1 (Figure S8b) and assayed it by transient expression using several promoters and activation complexes. Surprisingly, TFIIIA-7ZF co-expression influenced, significantly, the tripartite Myb2/bHLH2/WDR1 (MBW) [47] and bipartite WRKY1/WDR1 (WW) [48] complexes, activating chalcone synthases and WRKY promoters selected for this assay (Table S2). These results suggest that TFIIIA-7ZF has the potential to modulate or change expression of some genes upon viroid infection.

To demonstrate a possible direct influence of this factor to viroid replication, we performed co-expression of AFCVd with either TFIIIA-7ZF or control vector, followed by Northern blot analyses to check for viroid levels and distribution of replication intermediates of (+) polarity, which are well detectable for this viroid by molecular hybridization. A significantly higher accumulation of total (+) AFCVd in *N. benthamiana* was not observed after the co-expression with the TFIIIA-7ZF variant; however, in comparison to controls, there was a reproducible re-distribution and accumulation of hexameric (+) AFCVd intermediates. This band became apparently more abundant as counted from the distribution of radioactivity signal in the gels (Figure S9), while somewhat stronger signals were detected in gel zones expressing shorter replication intermediates of (+) polarity in the controls. These results suggest an involvement of TFIIIA-7ZF in some stabilization of AFCVd replication intermediates, and therefore, its lower level in pollen could have in principle some negative effect on AFCVd propagation.

## 3. Discussion

Many viroids are transmissible by seed and pollen, but certain viroid species are non-transmissible via pollen in certain hosts. A typical example of a viroid, which is non-transmissible via generative phases, is HLVd [31,49]. In the present work we analyzed the two viroids AFCVd and CBCVd infecting hop, as they are relatively young viroid isolates of economical significance that, unlike HLVd, are able to infect some common hosts from *Solanaceae*, such as *N. benthamiana* [33]. AFCVd and CBCVd showed no transmissibility via *N. benthamiana* seeds and thus are candidates for analysis of viroid elimination mechanism(s). For infectivity trials, we selected tobacco (*N. tabacum*) as an almost ideal model for plant reproduction and gametogenesis studies [50,51]; that is, the developmental stages of tobacco pollen can be defined according to lengths of buds and immature pollen can be separated by anther fractionation [34]. AFCVd infection of tobacco was achieved using biolistic inoculation with native viroid RNA as inoculum. Neither vertical nor horizontal AFCVd infection was observed in this new experimental host. There was, however, no blockage in the transport of AFCVd to anther tissues because the viroid levels in developing somatic anther tissues and young leaves were similar. Tobacco inoculation with CBCVd, either as native RNA or cDNA without promoter (or integrated into a plant vector), was unsuccessful (Table 1) obviously due to the inability of CBCVd to move in tobacco. However, transformation of tobacco with an infectious plant vector bearing dimeric CBCVd under 35S promoter initiated high viroid accumulation, including characteristic replication oligomers well visible on Northern blots; that is, this viroid can also replicate by a high rate in tobacco via the asymmetric rolling-circle mechanism. This enabled us to include CBCVd infection promoted by plant transformation for some comparative analyses.

Determination of AFCVd levels in leaves of infected tobacco and in different stages of pollen maturation clearly showed that there was a dramatic decrease in viroid signal on Northern blots: levels inside pollen were 4.5–3 × 10^5^ times lower than in leaves or somatic tissues (Figure 3). Only traces of viroid were finally detectable in growing pollen tubes roughly in the range of 5–50 × 10^−5^ per mg of pollen tissue. This suggests a dramatic depression of AFCVd propagation in pollen. These results are similar to observations by other authors who were able to detect only very low viroid levels or even no viroid in pollen; for example, ASSVd and DAVd were not detected in pollen from infected apple by PCR [16], and HLVd was detectable in hop pollen only by PCR-based methods [31]. Even in the case of PSTVd, which is in general vertically transmissible, the viroid was only detectable by in situ hybridization and sensitive RT-PCR in tomato pollen [21]. Recently the same research group [25,52] investigated tomato planta macho viroid (TPMVd), PSTVd and their synthetic chimeras; they described the disappearance of PSTVd and a TPMVd-based chimera containing the terminal-left (TL) domain of PSTVd with the elongation of pollen tubes in style and ovary. Specifically, the drop in PSTVd concentration resulted in a decline in the vertical transmission rate and the PSTVd concentration was below the detection limit of the RT-qPCR assay after pollination [52]. The authors concluded that factor(s) in the TL domain of PSTVd might be involved in the disappearance of viroids in pollen grains during the elongation process and the inability of viroids to be transmitted vertically [25]; however, the mechanism of viroid elimination from growing pollen tubes has not been analyzed. Unlike the PSTVd/tomato combination, we detected a significantly lower AFCVd propagation in comparison to leaves earlier, already at pollen developmental stage 3, when starch deposition begins and most of the vegetative cell is filled with cytoplasm. Viroid level was much lower than in somatic tissues and it dropped further to a very low level at stage 5 of pollen maturation, when the generative nucleus becomes spindle-shaped. Subsequently, the extremely low level in matured dormant as well as in early (6 h) germinating pollen suggested that the pollen germinating stage is the most important checkpoint for complete AFCVd elimination. From the germinating pollen stage, we were able to amplify full length AFCVd cDNA only after a strong concentration step of monomeric viroid using precipitation of 2 M LiCl-soluble nucleic acids.

We prepared tobacco transgenotes expressing analyzed viroids under the 35S promoter, which was weak in pollen, and under pLAT52, which was a strong promoter specific for the late pollen stages. These plant transformations led to increasing viroid expression and propagation in pollen, but the differences to viroid levels in leaves were still much higher than could be expected, providing an equal rate of native viroid machinery in both types of tissue. At the same time (−) strands of both viroid variants were clearly detectable by strand-specific RT-qPCR. Northern-blot analysis of pLAT52: AFCVd transformed/infected pollen clearly showed the presence of oligomeric replication intermediates, well comparable to spectra in leaves. These results suggest that, firstly, a block in trafficking was not the main obstacle for viroid propagation because a possible lack of movement and spreading within the population of maturating male gametophyte cells was overcome by the promoter support in transgenic tissue, and, secondly, that there was rather some deficiency or depression in the viroid replication pathway in pollen.

The replication pathway of *Pospiviroidae* members proceeds by an asymmetric rolling-circle mechanism known to be mediated by Pol II of the host [11]. Besides host Pol II and some of its regulatory elements, which are redirected to replicate viroid RNA, this mechanism involves presumably an RNase III-like enzyme for processing of (+) intermediates to monomers [53] and DNA ligase 1 for ligation of monomers to circles [54]. Monomeric and oligomeric AFCVd and CBCVd, respectively, were well detectable on corresponding Northern blots in pollen and in germinating pollen tubes, suggesting no significant deficiency in the processing step; even circular AFCVd was well detectable in AFCVd-infected stage 3 and transformed pollen tube samples, suggesting presence of the ligation machinery in these tissues.

Pollen as the male gametophyte fulfills a very specialized function connected with fast activation of proteosynthesis machinery during the first steps of pollen germination. Fast physiological processes such as pollen germination require an earlier production of mRNAs and their storage to ensure quick translation activation. Messenger RNAs that are transcribed during pollen development are stored in ribonucleoprotein particles (monosomes) in the cytoplasm and are quickly re-associated with polysomes after pollen hydration and germination [55]. Abundant proteosynthesis in pollen is connected to biogenesis of 5S rRNA involving RPL5 and TFIIIA. According to recent analyses [44,45,46] viroid replication is enhanced via TFIIIA-7ZF, a seven zinc-fingers containing variant of TFIIIA with nine zinc fingers [56,57]. We did not quantify directly levels of TFIIIA-7ZF, but relative levels of TFIIIA-(7ZF/9ZF) and RPL5 during pollen development favor a possible lack of TFIIIA-7ZF, which then could lead to reduced viroid replication. Relatively high level of TFIIIA-(7ZF/9ZF) mRNA was detected in healthy and infected tobacco leaves, lower levels in healthy and infected pollen stage 3, and very low levels in pollen at late stages (mature-dormant and germinating pollen). RPL5 mRNA was quite abundant in developing pollen stage 3, where it showed an even higher level than in leaves (Figure S11). The decreasing viroid level, due to degradation (see below), might successively lead to a pronounced imbalance of viroid/Pol II/TFIIIA-7ZF/TFIIIA-9ZF/RPL5 because of insufficient viroid to mediate optimized binding and expression of RPL5 for viroid propagation.

We cloned TFIIIA-7ZF mRNA from PSTVd-infected *N. benthamiana* and used it for co-expression with AFCVd. TFIIIA-7ZF, which is predominantely located in the nucleolus as well as in the nucleoplasm, can bind (−) PSTVd [58] and is a dedicated factor critical for Pol II-dependent PSTVd transcription to generate longer-than-unit-length products [45]. We did not detect a strong accumulation of AFCVd during TFIIIA-7ZF co-expession in *N. benthamiana* leaf sectors; there was, however, some redistribution of hybridizing viroid RNAs towards accumulation of hexameric (+) strands. This finding is in accordance with a possible role of TFIIIA-7ZF in driving the replication cycle to longer-than-unit-length products. With PSTVd, TFIIIA-7ZF binds to a region in the terminal left domain [45] mostly overlapping with the TCR, which is conserved between PSTVd and AFCVd (see Figure 1). Thus, the higher accumulation of AFCVd replication intermediates in TFIIIA-7ZF-overexpressing plants appears to support a function of TFIIIA-7ZF in AFCVd transcription. Moreover, our supplementary data (Table S2) show that TFIIIA-7ZF has the ability to interfere with various transcription complexes in the transient expression system. More analyses are necessary to clarify the possibility that this feature could be involved in viroid propagation and/or pathogenesis.

In RNA samples from AFCVd-infected and AFCVd and CBCVd-transformed/infected pollen, specific halos or comets hybridizing to viroid cDNA are visible on Northern blots of agarose and acrylamide gels (Figure 3, Figure 5 and Figure S2). These comets, which are degradation products with shorter-than-unit length according to size markers, were well visible especially during developmental stages 3 and 5, formed a significant signal in growing pollen tubes, and were more abundant in pollen than in leaves. We analyzed the viroid-specific degradation products using additional methods like acrylamide gel electrophoresis under denaturing conditions or under discontinuous pH gradient. This analyses showed discrete AFCVd fragments ranging from 300 to 100 nucleotide well detectable by molecular hybridization in stringent conditions. Obviously, the circular AFCVd is linearized and then cleaved to shorter products by endonucleolytic cleavage (Figure 5); that is, nucleolytic activities differing from the silencing machinery are involved in AFCVd degradation in pollen. This degradation was detectable in growing pollen tubes, suggesting that degradation mechanisms are part of the viroid cleaning machinery. We detected similar degradations of HLVd in hop pollen [31] and during thermotherapy of HLVd-infected hop mericlones [59], which is used to eradicate otherwise very resistant viroid infections [60]. PSTVd turnover and subgenomic PSTVd RNAs were recently characterized: the predominant cleavage was predicted to be caused by endoribonucleases not fully specific for single-stranded RNA producing 5’ OH and 3’ P termini [61]. In principle, such cleavage can be caused by S-like and E-like endoRNases or endoRNases from the T2 family expressed in pollen [31], and/or by the evolutionary conserved domain of TSN [62], producing 5’ OH and 3’ P termini, which are shared by plant TSN (for a review of plant TSN functions and biochemical properties, see [63]). TSN as a multifunctional enzyme forms part of mRNA processing bodies and stress granules [63]. In contrast, such cleavage cannot be mediated with pollen extracellular nuclease I, which produces 5’ P and 3’ OH ends [64]. While 5’ OH and 3’ P cleavage should efficiently inactivate viroid, 5’ P and 3’ OH ends can, in principle, be re-ligated by the redirected DNA ligase [54].

We analyzed vd-sRNA produced by the silencing machinery of the host. NGS data were initially derived from AFCVd-infected pollen stages 3 and 5 and from AFCVd-infected young tobacco leaves. We were not able to get enough clean reads from infected pollen tubes containing only traces of AFCVd. From plants transformed with LAT52:AFCVd, which contained about 1000 times more viroid in growing pollen tubes, pre-purification and NGS of small RNAs was successful. The number of vd-sRNAs originating from (−) strands was similar to that from (+) strands in leaves and in pollen; only in LAT52:AFCVd transformed/infected leaves were more (+) vd-sRNAs present (Figure 6 and Figure S5). A similar ratio of (−) to (+) vd-sRNA points to a critical preference of the silencing machinery for degradation of (−) strands, which were quantified by strand-specific RT-qPCR as much less abundant than (+) strands. It is not known, however, whether this preference by itself could influence the rate of viroid replication in pollen. The vd-sRNAs from pollen stages 3 and 5 contained a higher percentage of 5’ C than those from leaves. This points, in accordance with Minoia et al. [42], to a possible function of *Nt*AGO5 in AFCVd degradation in immature pollen.

A preliminary proteome analysis indeed showed some increase of factors in AFCVd infected pollen, which may be connected to viroid degradation. These factors included DCL enyzmes, AGOs and the nuclease TSN. Expressions of these selected candidate genes were quantified using RT-qPCR. mRNA of all of these factors showed high levels in pollen stage 3, while at later stages the expression significantly dropped to low, but still detectable levels in mature-dormant and germinating pollen (Figure 7). Therefore, these degradation factors can be expected to play some role in viroid elimination also at this stage providing the simultaneously low viroid replication rate in growing pollen tubes. According to our supplemental analyses of viroid levels in transformed/infected AFCVd and CBCVd mature-dormant and germinating pollen, there is a preference for degradation or/and low replication of (−) chains for both viroids (Figures S3 and S4). In case of CBCVd there was even a reversed ratio of (−)/(+) strands observed in comparison to leaves, where (−) chains prevailed over (+) chains in tobacco and also in some other species like hop and *N. benthamiana* [33]. In addition, overexpression of cloned *Nt*AGO5 from pollen in leaf sectors of CBCVd-pre-infected *N. benthamiana* plants led transiently to lower accumulation of (−) CBCVd chains, suggesting a possible influence of this AGO on the viroid replication cycle, although in the original work of Minoia et al. [42] there was no strand preference observed for AGO5. It has to be noted, however, that from three genomic homologues of AGO5 in *N. tabacum* only one variant (Figure S8) was significantly expressed during development of tobacco pollen (Figure 7).

## 4. Materials and Methods

### 4.1. Plant Cultivation Conditions, Pollen Stages Collection and In Vitro Pollen Germination

*N. tabacum* (cv. Samsun; Institute of Experimental Botany, CAS, Czech Republic) plants were grown under greenhouse conditions in Biology Centre, CAS, IPMB in České Budějovice, Czech Republic, in the seasons 2018 and 2019. Plants were intensively flowering from beginning of June to end of August. Opened flowers were removed daily to promote new flower buds. *N. benthamiana* plants were maintained in climate boxes at a temperature of 25 ± 3 °C with supplementary illumination (90 μmol/m^2^/s PAR) to keep a 16 h-day length.

Fresh flower buds of healthy, viroid-infected or viroid-transformed/infected *N. tabacum* of particular stage were collected according to their length and morphology [34]. Procedure of pollen stage isolation was carried out at 4 °C. Intact anthers were isolated from 20 flower buds (maximum per sample) to pre-chilled mortar. The anthers were gently cracked using mortar and pestle with 2 mL of 5% sucrose. The mixture was transfered to 50 mL falcon tube; additional 2 mL of 5% sucrose were used to rinse mortar; both mixtures were combined in the falcon tube and vortexed intermittently for 20 s to release pollen. The suspension was filtrated through nylon mesh to separate pollen from the anther debris. Pollen filtrate was divided into two 2 mL-tubes and centrifugated at 2000 g for 5 min at 4 °C. The supernatant was removed. The residual fine cell debris containing chloroplasts was removed manually from the top of the pollen pellet. Pollen pellet weight was determined and stored at −80 °C until analysis.

Isolated anthers from flower stage 6 (flowers one day before anthesis [34]), which were collected daily, were left on filter paper in Petri dish to dehisce on dry place overnight at room temperature. Nylon mesh was used to separate mature pollen from anther debris. Dry mature pollen was weighted and stored at −20 °C until analyses (in vitro pollen tubes cultivation, RNA extraction, microscopy, etc.).

Mature pollen was germinated in vitro in SMM-MES medium containing 175 mM sucrose, 1.6 mM H_3_BO_3_, 3 mM Ca(NO_3_)2·4H_2_O, 0.8 mM MgSO4·7H_2_O, 1 mM KNO_3_ and 25 mM MES pH 5.9 (modified from [65]). An aliquot of pollen (10 mg) was first incubated for 5 min at room temperature, vigorously vortexed in 1 mL of media for 4 min to allow pollen hydration and then the homogeneous suspension was transferred to 100 ml-Erlenmeyer flasks containing cultivation medium to a final volume of 10 ml. Pollen was cultivated at 1 mg/mL concentration for 6 h at 27 °C using a GLF 3032 shaker (Bahoefer, Germany) at 160 rpm for 3 h to prevent pollen sedimentation; then the speed was adjusted to 60 rpm and the cultivation continued for another 3 h. After 6 h cultivation, a 20 μL aliquot of pollen tube culture was taken prior to pollen tube mass harvesting for the measurement of pollen tubes length (see below Microscopy section).

To assay dynamics of pollen tube growth in mass culture, the “weight” method [65] was used; i.e., the culture was filtrated under mild constant pressure and immediately weighed. The weight corresponds to the volume of tubes and is a measure of growth activity of the culture [65]. Filtrated pollen mass culture was frozen at −80 °C until further analysis (isolation of RNA and proteins).

### 4.2. Hop Viroid Strains, Viroid Inoculation and Detection by Dot-Blot

For inoculation of *N. tabacum*, either AFCVd (GenBank Ahe C AB104533/clone AK6-3) or CBCVd (AC KM211547) were used [33]. Inocula were applied either as full-length copies of viroid cDNAs prepared as restriction fragments *Sac*I and *Sal*I, for CBCVd and AFCVd, respectively, or as native viroid RNA isolated from infected host plant *N. benthamiana*. Fragments of viroid cDNAs were inoculated using biolistic method [33]. Native RNA inoculum was prepared either as crude extract made in 0.04 M phosphate buffer pH 7.6 using mortar and pestle and applied on leaves using Carborundum as tabrasive, or RNA was isolated using Plant RNA Purification Reagent (Invitrogen, Carlsbad, CA, USA) followed by precipitation of nucleic acids with 2 M LiCl on ice for 1 h; LiCl-soluble RNA samples containing viroids were precipitated by ethanol and immobilized on gold microcarriers (1 μm) using a calcium-mediated precipitation protocol [66]. A Helios Gene Gun (Bio-Rad) was used for biolistic inoculation of cDNA or native RNA at a pressure of 130 psi at a distance of approximately 1 cm from the GeneGun spacer, as described previously for hop [33]. Each tobacco plant was inoculated five times in total with approximately 50 ng DNA or 100 ng of LiCl-soluble RNA per viroid species. In some experiments, we inoculated *N. benthamiana* plants using leaf infiltration with agrobacterium LBA4404 bearing infectious dimeric CBCVd or AFCVd vectors (Figure S1) according to a protocol used for transient expression [47]. RNA was extracted from *N. benthamiana* three weeks after infiltration. AFCVd clone AK6-3 was quite stable in *N. benthamiana*, as no mutated sequence variants were detected in a cDNA library. Dot-blot hybridization was primarily performed [67] using leaves tissue and full-length AFCVd and CBCVd ^32^P[dCTP]-labeled probes to check for AFCVd and CBCVd infections, respectively. Membranes from dot blots were scanned using the Typhoon PhosphoImager (Amersham Biosciences, Sunnyvale, CA, USA). The detection limit of this method is about 0.5 pg of viroid per dot [67].

### 4.3. RNA Extraction and Purification, RT-PCR, Strand-Specific Viroid RT-qPCRs and mRNA Quantification by RT-qPCR

One-hundred milligrams of extracted or fresh pollen or pollen mass culture was homogenized in 600 μL of Concert™ reagent (Plant RNA Purification Reagent, Invitrogen, Carlsbad, CA, USA) in prechilled mortar and pestle using sea sand. After transfer of the homogenate to Eppendorf tubes, RNA was further isolated according to manufacture’s protocol supplied for Concert reagent followed by RNA purification and a DNA cleavage step on columns (RNeasy Plant Total RNA kit, Qiagen, Hilden, Germany). Finally, supplemental DNA cleavage was performed with DNA-free™ DNA Removal Kit (Life Technologies, Brno, Czech Republic) for RT-qPCR analyses and viroid quantifications. The absence of any traces of DNA in the samples was checked by qPCR of viroids or target genes. This supplementary cleavage step was omitted for Northern blot analyses. For RNA extraction from plant leaves, a random mixture of cuttings from at least six young leaves was used. If not stated otherwise, 100 mg of tissue from this random mixture was used for extraction using standard protocol with Concert reagent followed by RNA purification. In some experiments we concentrated viroid RNAs from pollen tubes using a combination of Plant RNA Purification Reagent, followed by isolation of 2 M-LiCl-soluble fraction that was then precipitated with ethanol and processed by DNA cleavage steps. For isolation of small RNA from leaves, developing pollen and transformed pollen tubes for NGS, we used the protocol for Plant RNA Purification Reagent in combination with separation of small RNAs on columns of PureLink™ miRNA Isolation Kit (Thermo Fisher Scientific, Brno, Czech Republic).

For full-length AFCVd amplification and construction of a cDNA library, RT-PCR was performed using the Titan One Tube RT-PCR (Sigma-Aldrich, Prague, Czech Republic) including a high fidelity *Pwo* polymerase (Sigma-Aldrich, Prague, Czech Republic). Primer combination AFCVd IIsa × AFCVd Isa (numbers 17 and 18 in Table S1) were used for full length AFCVd clones [33]. Full viroid copies were cloned into the vector pCR-Script SK(+) (Stratagene) and sequenced by Eurofins Genomics [68].

For simultaneous RT-qPCR quantification of viroid (+) and (−) strands, we used a singlestrand-specific RT-qPCR method described previously [33]. Briefly, this procedure uses two sets of viroid specific primers and the highly thermostable *Tth* polymerase to disrupt the highly stable viroid RNA structures. In the first step, we used *Tth* polymerase for reverse transcription of viroid RNA with (+) AFCVdRTPL, CVdRTPL (numbers 13 and 25 in Table S1) or (−) AFCVdRTMI, CVdRTMI (numbers 14 and 26 in Table S1) RNA at high temperature, and, in the second step, real-time PCR quantification was performed with the cDNA using PCR FOR and PCR REV primer combinations (numbers 15 × 16 and numbers 27 × 28 in Table S1) for AFCVd and CBCVd, respectively. These PCR reactions led to amplification of a 81 and 85 bp product for AFCVd and CBCVd, respectively. For the first step, 1 μg of purified and DNAse-treated total RNA was mixed with 20 pmols of either RTPL or RTMI primer in a reaction mixture containing MnCl_2_ for reverse transcription using *Tth* polymerase (5μU/μL, Promega, Madison, WI, USA) with a hot start and run for 30 min at 70 °C [33]. Five microliters of each 20× diluted RT sample was added, and 10 μL of “iQ™ SYBR^®^ Green Supermix” (Bio-Rad). The quantification was performed on the CFX Connect™ Real-Time PCR Detection System (Bio-Rad) with Bio-Rad CFX Maestro qPCR software v1.1. After the initial denaturation at 94 °C for 4 min, 40 amplification cycles (94 °C/20 s, 61 °C/40 s, 72 °C/30 s) were performed. Relative viroid levels were normalized with the “delta-delta method” [69] to the levels of 7SL RNA [29,33,70]. Because of similar structures of 7SL RNA and viroid, the same thermostable RT system was used (with 7SL α and anti-β primers, numbers 51 and 52 in Table S1). In some experiments, we ran viroid and 7SL RNA together as a duplex reaction (see Figure legends). For statistical analysis we calculated *p*-values for level of significance using the two-tailed *t*-test as in previous analyses [33]. To evaluate the efficiency of the qPCR reaction for each set of primers, five different concentrations of viroid DNA template were analyzed in duplicates to obtain a standard curve. The assay had an efficiency of 99% for AFCVd, 95% for CBCVd and 107% for corresponding primers.

Quantification of mRNA levels of genes potentially connected to viroid degradation selected from developing pollen was performed according to a published protocol [33] with slight variations in annealing temperatures. The primer combinations listed in Table S1 for quantification of mRNA levels were derived based on multiple alignments of *N. tabacum* and *N. benthamiana* sequences identified via BLAST in GenBank and in *Nicotiana* genomes [71,72]. The following mRNAs were quantified with primer annealing temperatures (across slashes): *Nt*1AGO5/55.6 °C, *Nt*TUDOR S-like nuclease (TSN)/58 °C, *Nt*DCL homologues/54 °C, *Nt*TFIIIA/56 °C, *Nt*RPL5/55 °C, GFP/58 °C and *At*bZIP18/55 °C. Note that the*Nt*DCL primers do not differentiate between DCL1–4. Results were normalized to *Nt*ACT/51 °C as internal control. One μg of purified and DNAse-treated total RNA was reverse transcribed using oligo dT18 primer and SuperScript™ III reverse transcriptase (Invitrogen, Carlsbad, CA, USA) at 50 °C for 60 min. RT-qPCR was then performed on the CFX Connect™ Real-Time device (Bio-Rad, Hercules, CA, US) using 20 μL of reaction mixture containing 5 μL of 20-fold diluted cDNA, 5 μL of 2 μM forward and reverse gene-specific primers (Table S1) and 10 μL 2× SYBR™ Green PCR master mix (Applied Biosystems), under the following amplification condition: initial denaturation at 95 °C for 3 min, followed by 40 cycles of denaturation at 95 °C for 30 s, annealing at temperatures as above for 30 s and extension at 72 °C for 35 s. At the end of the reaction, the specificity of each primer pair was assessed using a melting curve analysis. Product sizes were confirmed by melting analysis and 2% agarose gel electrophoresis. The abundance of a reference transcript of *Nt*actin was estimated in parallel in each sample. $C_{t}$ values were measured using CFX Maestro qPCR software v.1.1 (Bio-Rad). The relative values were standardized with the delta-delta method and normalized to the sample with germinating pollen, where the calibrator was set to 100%. The data points show the means ± SD of two replicates of each PCR reaction.

### 4.4. Northern Blot Analyses in Agarose and Acrylamide Gels and Molecular Hybridization

For AFCVd and CBCVd Northern blot analyses, RNA was electrophoresed on 1.5% formaldehyde- denaturating agarose gel and blotted onto Biodyne A transfer membrane (Pall, Hampshire, England) by capillary blotting. Prehybridization and hybridization were carried out in 50% formamide-based (pre)hybridization buffer [59] at 55 °C. If not otherwise stated, samples were hybridized to [^32^P-dCTP]-labeled full-length viroid cDNA probes. The final washing was performed in 0.1 × SSC plus 0.1% SDS at 60 °C for 20 min. In some experiments, we used strand-specific RNA (−) or (+) probes. [$\alpha {}^{32}$P-UTP]-labeled RNA strands were transcribed from viroid templates having attached T7 promoters. Corresponding templates were prepared using primer numbers 19–22 and 29–32 (Table S1) for AFCVd and CBCVd, respectively.

For detection of AFCVd degradation products, AFCVd samples were electrophoresed in 12% acrylamide gels containing 30:1 acrylamide:bisacrylamide (w/w), 3 M urea, 17.8 mM Tris-borate and 0.048 mM EDTA (0.2 × TBE). To analyze for the presence of circular AFCVd, the acrylamide gel electrophoresis in a discontinuous pH gradient was performed essentially according to [73] except for gel concentration. Nucleic acids were run in 8% acrylamide gels containing 30:1 acrylamide:bisacrylamide (w/w) and 8 M urea. Nucleic acids were transblotted from acrylamide gels onto Nylon membranes (Charge Modified 0.2 μm; Sigma-Aldrich, Prague, Czech Republic) using a Panther™ Semidry Electroblotter (Owl Separation Systems, Portsmouth, NH, USA). Blotting was performed in 1× TBE buffer (89 mM Tris base, 89 mM boric acid, 2 mM Na_2_EDTA, pH 8.3) with a current of 2 m/A cm^−2^ of gel for 1 h. Afterwards membranes were UV-irradiated using a transilluminator and baked for 20 min at 80 °C. Hybridization was performed in 50% formamide hybridization buffer as described [59] to [$\alpha^{32}$P]UTP-labeled riboprobes at 50 °C overnight. The washing procedure included an incubation of membranes in 2× SSC for 10 min at 0 °C with subsequent washing. The final washing was performed in 0.25× SSC ±0.1% SDS at 55 °C for 20 min.

### 4.5. Molecular Cloning, Preparation of Plant Vectors, Plant Transformation and Transient Expression System

We found a weak expression of the 35S promoter in pollen (see Results); thus an alternative vector was developed based on pBIN-GFP [74]. Into this plant vector the polylinker *Hin*dIII·*Sma*I·*Pac*I·*Xho*I·*Kpn*I·*Asc*I·*Sda*I was first inserted within the T-DNA at positions 4950–4961 using the unique restriction sites *Hin*dIII and *Sda*I. Oligos 43 and 44 (Table S1) were used to prepare the polylinker. In restriction sites *Pac*I and *Xho*I within this polylinker the chalcone synthase promoter pchs_H1 [47] was integrated based of primers 45 and 46 providing *Pac*I and *Xho*I restriction ends to the promoter sequence (Table S1). The new expression cassette was then completed by integration of the *Xho*I/*Asc*I fragment from vector pRT-100 [75] providing the CaMV terminator. We named the resulting plant vector pJM14.

We amplified full-length cDNA of *Nt*AGO5 from AFCVd-infected pollen of *N. tabacum* stage 3 using primers 1AGO5Start and 1AGO5Stop (numbers 1 and 2 in Table S1), and *Nt*TFIIIA-7ZF cDNA from PSTVd-infected *N. benthamiana* using primers 7Zstart and 7Zstop (numbers 7 and 8 in Table S1). Both these sequences, re-amplified by *Pwo* polymerase, were first blunt-end cloned into pPCR-Script Amp SK(+) (Stratagene). Subsequently, *Xho*I,*Kpn*I and *Xho*I,*Bam*HI restriction ends were added to *Nt*AGO5 and *Nb*TFIIIA-7ZF using primers 3,4 and 9,10 (Table S1), respectively. *Nt*AGO5 cDNAs was then cloned directly into the chs_H1(600) promoter cassette of binary vector pJM14 (see above). *Nb*TFIIIA-7ZF was first cloned into pRT-100 via unique *Xho*I and *Bam*HI sites [75] and then re-cloned together with the terminator sequence into pJM14 using *Xho*I and *Asc*I. Finally, both *Nt*AGO5 and *Nb*TFIIIA-7ZF bearing plant vectors were transformed into *A. tumefaciens* LBA4404 and used for transient expression assays.

For analyses of promoter activation and for transient expression of factors in *N. benthamiana* leaves, co-infiltrations with *A. tumefaciens* LBA4404 containing various plant expression vectors bearing activation complexes [47,48] were performed using a GUS activating system described earlier [76]. The amount of the released fluorescent dye 4-methylumbelliferone (MU) was measured using the VersaFluor™ fluorometer (Bio-Rad) with excitation at 365 nm and emission at 455 nm. GUS activity was expressed in pmolMU/mg fresh tissue/min. At least three independent experiments were performed in each experimental variant. The data were statistically analyzed using Microsoft Excel 2010.

For plant transformation with infectious AFCVd dimer under pollen-specific promoter pLAT52, we modified plant vector pFASTbZIP18 [77] (Figure S1b) as follows: we used specific adapter primers Xho_ada_Kpn and Xba_ada_KpnI (numbers 23 and 24; Table S1) to amplify with the high fidelity *Pwo* polymerase the whole dimeric AFCVd as single *Kpn*I fragment using the 35S:AFCVd vector as template (Figure S1a). This fragment was then inserted into the unique *Kpn*I site of pFASTbZIP18 vector in position 6804 between 3’ end of sequence of bZIP18 and NOS terminator and selected as dimeric (++) AFCVd construct (Figure S1b) for tobacco transformation. Transformation of tobacco with AFCVd and CBCVd plant infectious vectors was performed using the standard leaf disc method [78]. Regenerated transformed plants were maintained on medium containing 100 mg l^−1^ kanamycin and 200 mg l^−1^ timentin.

### 4.6. Bioinformatic Methods and RNA Profiling

Sequence comparisons were carried out with DNASIS v2.6 (Hitachi Software Engineering Company, Tokyo, Japan). Protein domain sequence analyses were performed using InterProScan module of Geneious Prime v2019.04.

Small RNAs were sequenced by LCSciences (Houston, TX, USA) and libraries were registered with the BioProject database (ID PRJNA610504 [79]). Reads were trimmed by trimmomatic [80], mapped with bowtie v1.2.2 [39] to the AFCVd sequence of increased length (1–371/1–24) allowing for one error. Mapping reads first to the *N. tabacum* genome and the resulting unaligned reads to the AFCVd sequence resulted in no difference compared to the direct mapping to AFCVd. Output was parsed by a perl script and drawn by GLE [81].

### 4.7. Pollen Microscopy

A small aliquot of tobacco mature pollen was resuspended in DAPI staining solution for cell nuclei visualisation (16 μL of DAPI stock solution in 10 mL of extraction buffer, modified according to [82]). Bright field and fluorescence (UV light) microscopy were used for phenotype analysis of tobacco pollen on an inverted fluorescent microscope Nikon Eclipse TE2000U. Three pollen phenotype categories were evaluated: mature and late bicellular pollen with spindle-shaped generative nucleus and diffuse vegetative nucleus; collapsed pollen without any visible nuclei; early bicellular pollen with rounded generative nucleus and diffuse vegetative nucleus. The percentage of individual pollen phenotype categories was calculated from at least 150 pollen grains and three replicates per tobacco line.

Images of 6 h pollen tubes in bright field were taken to measure the length of individual pollen tubes (minimum of 150 pollen tubes and three replicates per tobacco line) using NIS-Elements (Nikon Imaging Software, Amsterdam, Netherlands) for image analysis.

### 4.8. Protein Extraction and Filter-Aided Sample Preparation

Samples of pollen stages 3 and 5, mature pollen and pollen tubes from individual tobacco lines in three replicas were homogenized in the mortar using liquid nitrogen. TRI Reagent solution (Sigma-Aldrich, Prague, Czech Republic) was used for protein isolation. Extracted proteins were processed using filter-aided sample preparation (FASP) protocol [83,84] using Microcon 30-kD filter unit (Millipore; MILLMRCF0R030), including alkylation step (iodoacetamide). Trypsin (sequencing grade; Promega, Madison, WI, USA) was used for protein digestion (overnight at 37 °C). The resulting tryptic peptides mixture was cleaned by liquid-liquid extraction using ethylacetate (3 iterations) [85].

### 4.9. LC-MS/MS Analysis

LC-MS/MS analyses of all peptide mixtures were done using RSLCnano system connected to Orbitrap Q Exactive HF-X spectrometer (Thermo Fisher Scientific). The tryptic digests were online concentrated and desalted on a trapping column (Acclaim™ PepMap™ 100 C18, dimensions 300 μm × 5 mm, 5 μm particles) and eluted (300 nl/min) onto an analytical column (Acclaim Pepmap100 C18, 3 μm, 75 μm × 500 mm; Thermo Fisher Scientific) by 130 min gradient program (2–35% of mobile phase B; A: 0.1% FA in water; B: 0.1% FA in 80% ACN). Digital PicoView 550 (New Objective) ion source with ABIRD (Active Background Ion Reduction Device, ESI Source Solutions) was used.

MS data were acquired in a data-dependent strategy (top20; survey scan 350–2000 m/z, resolution 120,000, target value 3 × 10^6^, maximum injection time 50 ms). HCD MS/MS (27% relative fragmentation energy) spectra were acquired (target value 10,000, resolution 15,000, maximum injection time 50 ms, isolation window 1.2 m/z) with dynamic exclusion enabled for 40 s after one MS/MS spectra acquisition.

The analysis of the mass spectrometric RAW data files was carried out using the MaxQuant v1.6.2.10 software using default settings. MS/MS ion searches were done against modified cRAP database (based on [86], and tobacco protein database ([87]; version from 13.4.2017, number of protein sequences 69,500). Oxidation of methionine and proline, deamidation (N, Q) and acetylation (protein N-terminus) as optional modification, carbamidomethylation (C) as fixed modification. Match between runs was set among all analyzed samples. Protein intensities reported in proteinGroups.txt were further evaluated using the software container environment ([88]; version 3.7.2a).

## 5. Conclusions

**Figure 8:**
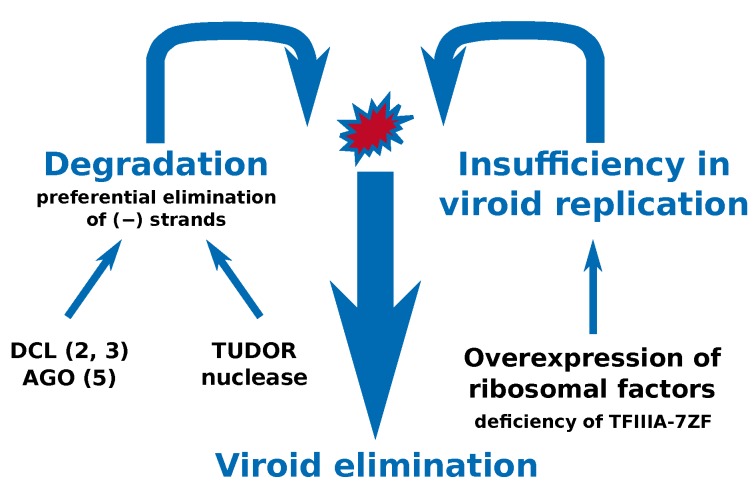
Schematic drawing of two major processes–the degradation and depression of a viroid replication pathway, participating in the elimination of this parasitic RNA.

We propose a model (Figure 8) that underlines the findings that two basic processes have to occur simultaneously to shift the viroid turnover to significant depression or complete viroid elimination. We assume that these processes include, firstly, a depression of the viroid replication machinery possibly due to lower levels of Pol II-assisting or auxiliary factors such as TFIIIA-7ZF and RPL5, and secondly, an increase in viroid degradation. The degradation part of this model could involve viroid-protecting factors—RNA and viroid-binding proteins that have already been considered in relation to viroid elimination from pollen [31]. Here we showed that there are several modes of nucleolytic attack on viroid propagating in pollen by the RNA degradation factors, including silencing, and obviously less specific nucleolytic activities connected to cellular RNA turnover with the ability to destroy the otherwise strongly resistant circular viroid form and structured viroid RNA. By forcing a non-pollen-transmissible viroid to be expressed via transgenosis in pollen, we showed in *N. tabacum* that AFCVd elimination is not interconnected to strong pathogenic physiological reactions of pollen.

## Figures and Tables

**Table 1:** AFCVd and CBCVd infection tests on *Nicotiana tabacum*.

Viroid	Inoculum	Inoculation Method	Number of Plants	
			Inoculated	Infected ${}^{a}$
AFCVd	Crude RNA from *N. benthamiana*	Carborundum	12	1
AFCVd	Full length genomic restriction fragments *Sal*I	biolistic ${}^{b}$	12	0
AFCVd	RNA (native viroid ${}^{c}$) from *N. benthamiana*	biolistic ${}^{b}$	12	9
CBCVd	Crude RNA from *N. benthamiana*	Carborundum	12	0
CBCVd	Full length genomic restriction fragments *Sac*I	biolistic ${}^{b}$	12	0
CBCVd	RNA (native viroid ${}^{c}$) from *N. benthamiana*	biolistic ${}^{b}$	12	0

${}^{a}$ Dot-blot analysis was performed two months post inoculation (Figure 2). ${}^{b}$ Biolistic inoculation was performed: each plant was shot four times with either 100 ng of RNA or 50 ng of cDNA. ${}^{c}$ Pre-purified 2 M-LiCl fraction of RNA for preparation of carrier.

**Table 2:** Analysis of phenotypes and vitality of pollen collected from healthy and viroid-infected tobacco genotypes.

	Parameters of Vitality ${}^{a}$			
Genotypes	Young Bicellular [% ± SD]	Aborted Pollen [% ± SD]	Mass of Pollen Tubes [mg ± SD]	Length of Pollen Tubes [μm ± SD]
AFCVd-infected ${}^{b}$	n.d. ${}^{c}$	2.45 ± 0.95	6.75 ± 0.71	1027 ± 362
Control ${}^{b}$	n.d.	3.87 ± 0.85	6.13 ±1.21	877 ±386
35S:AFCVd/infected ${}^{d}$	n.d.	9.77 ± 3.28	9.05 ± 0.84	895 ± 257
Control ${}^{d}$	n.d.	3.87 ± 0.85	6.95 ± 0.36	1084 ± 405
LAT52:AFCVd/infected ${}^{d}$	2.51 ± 0.57	6.32 ± 1.34	8.27 ± 2.19	999 ± 328
Control ${}^{d}$	2.05 ± 1.41	3.74 ± 1.15	10.52 ± 3.05	948 ± 300
35S:CBCVd/infected ${}^{d}$	1.17 ± 0.46	6.19 ± 2.05	6.30 ± 0.37	784 ± 273
Control ${}^{d}$	0.94 ± 0.41	3.68 ± 1.40	7.07 ± 0.74	1040 ± 325

${}^{a}$ Mass of pollen tubes germinating from 1 mg of mature pollen. See Figure 4 for pollen phenotypes. Mean values ± SD are given. ${}^{b}$ Non-transformed tobacco, AFCVd infected with the biolistic method; comparison with healthy controls. ${}^{c}$ Not determined. ${}^{d}$ Transformed/infected plants with the corresponding infectious plant vectors are shown; comparison with healthy controls.

## Supplementary Materials

The following are available online at https://www.mdpi.com/1422-0067/21/8/3029/s1: Table S1: Primers used for cloning, transcription templates, modification of plant vectors, strand-specific RT-qPCR and quantification of mRNA levels by RT-qPCR; Table S2: Influence of TFIIIA-7ZF on complex activation of promoters in transient expression system; Figure S1: Plant vector cassettes used for transformation of *N. tabacum*; Figure S2: Northern blot analysis of CBCVd intermediates; Figure S3: Comparative analysis of levels of AFCVd strands in leaves versus mature and germinating pollen in plants transformed with pLAT52:AFCVd vector; Figure S4: Comparative analysis of CBCVd (+) and (−) RNA levels in leaves versus developing (stage 3 and 5), mature and germinating pollen in plants transformed with 35S:CBCVd vector; Table S3: Overview of all smallRNA sequence data sets before and after the preprocessing; Figure S5: Mapping of vd-sRNA to the AFCVd genome and length distribution of vd-sRNA; Table S4: Percentage of AFCVd mutations according to NGS; Figure S6: Position of mutations in the AFCVd genome according to vd-sRNAs; Figure S7: 5’ end nucleotide of vd-sRNAs; Figure S8: Protein domains of two factors cloned and analyzed in transient expression systems in this study; Figure S9: Spectrum and semiquantitative distribution of AFCVd (+)-genomic chains representing monomeric viroid and (+) replication intermediates in control-infected *N. benthamiana* leaf discs infiltrated with empty vector pJM14 or after co-expression of factor TFIIIA-7ZF; Figure S10: Levels of CBCVd (+) and CBCVd (−) RNA in leaf sectors of pre-infected *N. benthamiana* plants infiltrated in leaf halves either with control plant vector pJM14 or with *Nt*AGO5 cloned from developing viroid-infected pollen; Figure S11: Relative mRNA levels of *Nt*TFIIIA and *Nt*RPL5 that could interfere with viroid replication cycle and/or viroid propagation in pollen.
