# Supplementary material for: Elimination of Viroids from Tobacco Pollen Involves a Decrease in Propagation Rate and an Increase of the Degradation Processes
Source: Int J Mol Sci. 2020 Apr 24;21(8):3029. doi: 10.3390/ijms21083029 (PMC7216239; doi:10.3390/ijms21083029)
Supplement: Supplementary file 1 [file ijms-21-03029-s001.pdf]

Supplementary Materials: Elimination of Viroids from Tobacco Pollen Involves a Decrease in Propagation Rate and an Increase of the Degradation Processes

Jaroslav Matoušek [1], Lenka Steinbachová [2], Lenka Záveská Drábková [2], Tomáš Kocábek [1], David Potěšil [3], Ajay Kumar Mishra [1], David Honys [2], and Gerhard Steger [4]*

Table S1. Primers used for cloning, transcription templates, modification of plant vectors, strand-specific RT-qPCR and quantification of mRNA levels by RT-qPCR.

No	Designation	Sequence 5′→3′	Purpose	Ref.
		***Nt*AGO5**		
1	1AGO5Start	ATGTCGGAACGTGGACG	cDNA cloning	
2	1AGO5Stop	TTAGCAATAAAACATGACATCC		
3	1AGO5XHO	AACTCGAGATGTCGGAACGTGGACG		
4	1AGO5KPN	TTGGTACCTTAGCAATAAAACATGACATC		
5	1AGO5F	CAGCCTTCATCATCACAACG	RT-qPCR	
6	1AGO5R	CGTCCAACAGTTCCGTATCC		
		***Nb*TFIIIA-7ZF**		[1]
7	7Z start	ATGCAAGAGAGGCCATTTG	cDNA cloning	
8	7Z stop	TCACAGCTCTTCATCTGATTCTG		
9	7Z Xho	AACTCGAGATGCAAGAGAGGCCATTTG		
10	7Z Bam	AAGGATCCTCACAGCTCTTCATCTGATTCTG		
		***Nt*TFIIIA**		
11	NtTFIIIA_F	GGAAAGATGAGGCAAATGAAG	RT-qPCR	
12	NtTFIIIA_R	CTTGGTGCTGCAAGAGATGTC		
		**AFCVd**		
13	AFCVdRTPL	TCGTCGACGACGAGTCACCAGGTG	cDNA synthesis &	[2]
14	AFCVdRTMI	GTGACTCGTCGTCGACGAAGGGTC	reverse transcription	[2]
15	AFCVd PCR FOR	CCGGTCGTGGATACCTAGGA	RT-qPCR	[2]
16	AFCVd PCR REV	ACGCGGCCTTCGGTGTG		[2]
17	AFCVd IIsa	CGTCGACGAAGGGTCCTCAG	Reverse transcription	[2]
18	AFCVd Isa	TTCGTCGACGACGAGTCACC	& cDNA library	[2]
19	AFCVd start	TGGGCTCCAACTAGTGGTTC	cDNA amplification &	
20	AFCVd T7 start	TAATACGACTCACTATAGGGCTCCAACTAGTGGTTCC	with T7 promoter for	
21	AFCVd stop	GGGCACCCAAACAAGGG	strand-specific RNA	
22	AFCVd T7 stop	TAATACGACTCACTATAGGGCACCCAAACAAGGG	probes	
		**pFASTbZIP18 adaptor**		
23	Xho_ada_Kpn	AAGGTACCGTTCATTTCATTTGGAGAGGACCTCG	modification of	
24	Xba_ada_KpnI	TTGGTACCAGAGACTGGTGATTTGCGGACTCTAG	pFASTbZIP18	
		**CBCVd**		
25	CVdRTPL	AAGCCTGGGAGGAACAACCCAAGAG	cDNA synthesis &	
26	CVdRTMI	GGATCCCCGGGGAAATCTCTTCAG	reverse transcription	[2]
27	CVd PCR FOR	TCACTGGCGTCCAGCACC	RT-qPCR	[2]
28	CVd PCR REV	AGGAAGAAGCGACGATCGG		[2]
29	CVd start	CTGGGGAATTTCTCTGCG	cDNA amplification &	[2]
30	CVd T7 start	TAATACGACTCACTATAGGGAATTTCTCTGCG	with T7 promoter for	[2]
31	CVd stop	GGGTCTCAAAAGCGGGC	strand-specific RNA	[2]
32	CVd T7 stop	TAATACGACTCACTATAGGGTCTCAAAAGCGGGC	probes	[2]
33	CVds I	AAGAGCTCCTGTTCCGGTGCTG	reverse transcription	[2]

continued on next page

Table S1 – continued from previous page

No	Designation	Sequence 5′→3′	Purpose	Ref.
34	CVds II	AAGAGCTCGTCTCCTTCCTTCC		[2]
		***Nt*RPL5 tobacco homologues**		
35	NtRPL5-F	ATGGCATTCATCAAAGTCCAG	RT-qPCR	
36	NtRPL5-R	GTGATGCATAAGCTGATGAGCG		
		***Nt*DICER-like homologues**		
37	pollenDcl-F	GAGTGCATGAAACATATGATACAG	RT-qPCR	
38	pollenDcl-R	GAGAACTCTCAAGAAGCMTTGA		
		***At*5bZip18**		[3]
39	At5Zip18-F	CATCATCATCAGCAACAAACCG	RT-qPCR	
40	At5Zip18-R	GTGATGCATAAGCTGATGAGCG		
		**GFP**		[4]
41	GFP-F1	CAAGAGCGCCATGCCTGAG	RT-qPCR	
42	GFP-R1	CGTGTCTTGTAGTTCCCGTCGTC		
		***Nt*TUDOR S1-like nuclease (*Nt*TSN)**		
43	TunucF	GTGGATGAGCCATTTGCATG	RT-qPCR	
44	TunucR	GATGCCTCAGAAGCACCAGG		
		**HS link**		[4]
45	HS link sens	TAAGCTTCCCGGGTTAATTAACTCGAGGTACCGGCGCGCCTGCAGGAA	Plant vector pBin-35S-GFP5	
46	HS link anti	TTCCTGCAGGCGCGCCGGTACCTCGAGTTAATTAACCCGGGAAGCTTA	modification	
		***Hl*chs_H1 promoter 600**		[5]
47	PchsH1Pac	AATTAATTAAGATCACGACCGTCCATTC	Construction of vector pJM13 and pJM14	
48	Pchs1endXho	AACTCGAGCATTTTTCCTTTAGTTTCGGA		
		***Nt*ACTIN**		
49	ACT-F1	TTCTGTTCCAACCATCAATGA	RT-qPCR	
50	ACT-R1	GTACCACCACTGAGGACAATGT		
		**7SL RNA**		[6]
51	primer α	TGTAACCCAAGTGGGGG	Reverse transcription	
52	anti-β	GCACCGGCCCGTTATCC	and RT-qPCR	

Table S2. Influence of TFIIIA-7ZF on complex activation of promoters in transient expression system.

**Promoter**	**Activators**	**Activation units**[a] (pmol of MU / min / mg)	**Activation/suppression levels with TFIIIA-7ZF** (%)[b]
pCHS_H1 1000[c]	WW[d]	69 ± 0.1	121
	M2BW[e]	1091 ± 118	83
	WW+p19[df]	3490 ± 713	91
	M2BW+p19[ef]	5937 ± 1989	87
pCHS4[g]	WW[d]	27 ± 1.6	162
	M2BW[e]	7 ± 0.4	79
	WW+p19[df]	1142 ± 87.9	254
	M2BW+p19[ef]	227 ± 10.1	140
pWRKY1[c]	WW[d]	24 ± 1.1	108
	M2BW[e]	3 ± 0.5	100
	WW+p19[df]	422 ± 9.6	63
	M2BW+p19[ef]	44 ± 1.4	149

[a] GUS activation units were determined by three independent measurements, as described in Material and Methods, 3.5 days after co-infiltration of promoter and activators in young *N. benthamiana* leaves. The numbers indicate pmol of MU per min per 1 mg of fresh tissue ±SD.
[b] Activation or suppression was determined after co-expression of bacterial strain bearing TFIIIA-7ZF with promoters and activators in the transient expression system; GUS activity level without TFIIIA-7ZF was set to 100 %.
[c] Promoter pCHS_H1 in hop was described by Matoušek et al. [5].
[d] Activation complex WW (*Hl*WRKY1/*Hl*WDR1) was described by Matoušek et al. [7].
[e] Activation complex M2BW (*Hl*Myb2/bHLH2/*Hl*WDR1) was described by Matoušek et al. [5].
[f] p19 is a silencing suppressor described e. g. by Baulcombe and Molnár [8].
[g] Promoter pCHS4 in hop was described by Matoušek et al. [5].

a

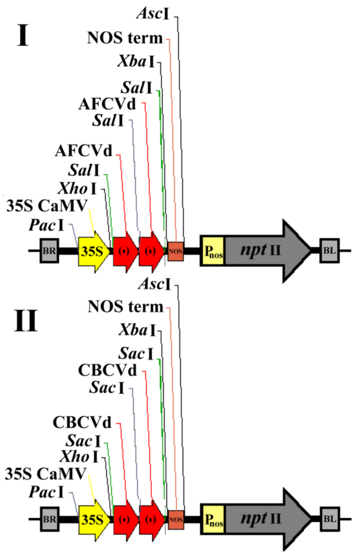

b

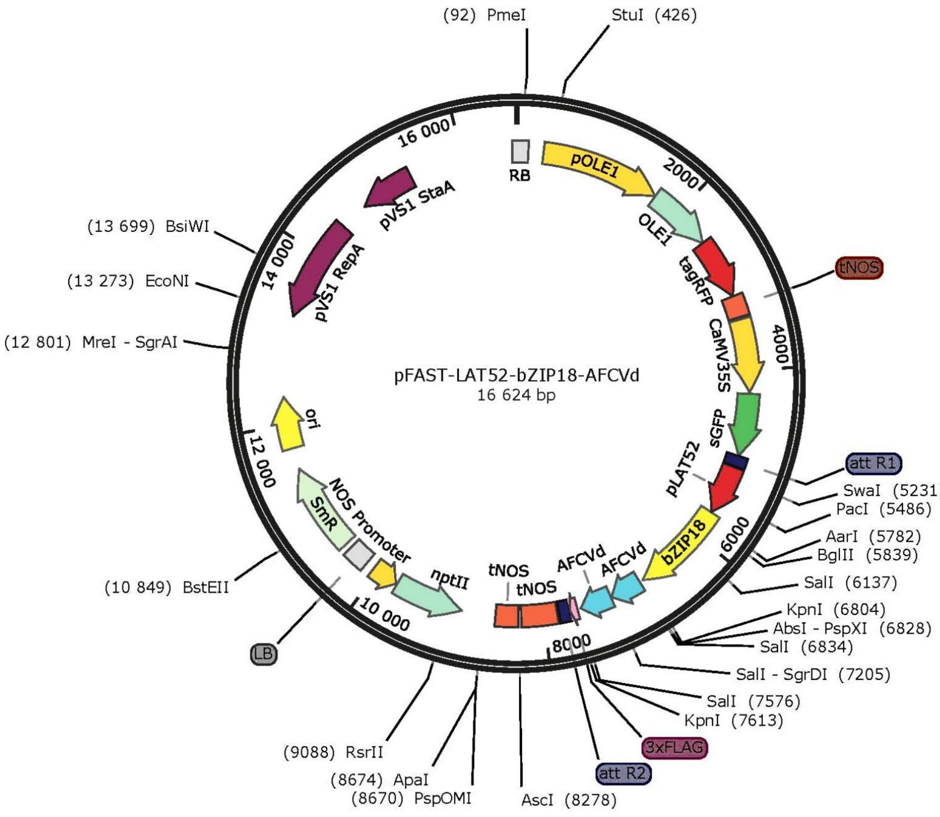

Figure S1. Plant vector cassettes used for transformation of *N. tabacum*. (a) AFCVd (I) and CBCVd (II) infectious dimeric (++) cDNAs of corresponding viroids integrated in modified expression cassettes containing CaMV 35S promoter and NOS terminator that were constructed as described previously [7] and cloned via *Pac*I and *Asc*I to plant vector pLV07 [7]. (b) Plant vector pFAST bearing late pollen-specific promoter pLAT52. Infectious (++) dimer of AFCVd is integrated via specific adapter (Table S1) in unique *Kpn*I restriction site downstream of bZIP18 gene under pLAT52. The final vectors were transformed to *Agrobacterium* LBA4404 and used for transformation. The schemes are not in scale.

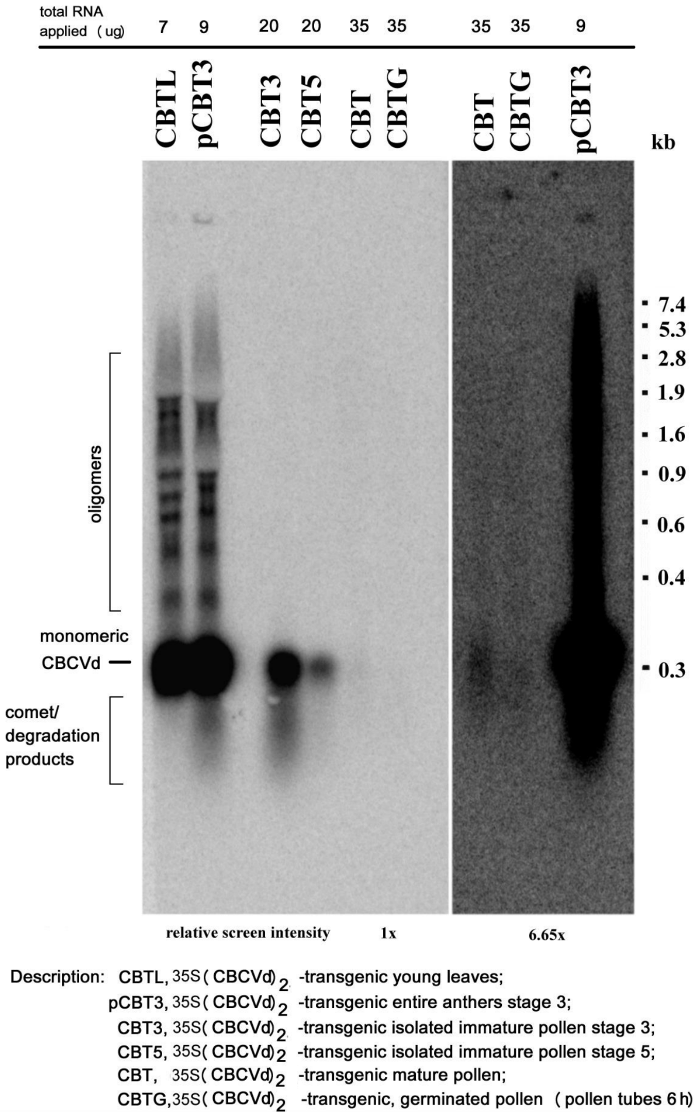

Figure S2. Northern blot analysis of CBCVd intermediates. Total RNA isolated from 35S:CBCVd-transformed tobacco leaves, anthers and pollen in amounts as indicated in the figure was electrophoresed in denaturing 1.5 % agarose gel and capillary transblotted to Biodine A Nylon membrane. Samples were hybridized to [^{32}P-dCTP]-labeled CBCVd cDNA as described in Material and Methods. Membranes were scanned after 24 h exposure, relative screen intensification is given in the bottom of the figure. The positions of CBCVd monomers, longer-than-unit-length RNA and comet degradation products are indicated on the left of the panel. Positions of RNA III marker (Boehringer Mannheim) is indicated on the right side of the panel.

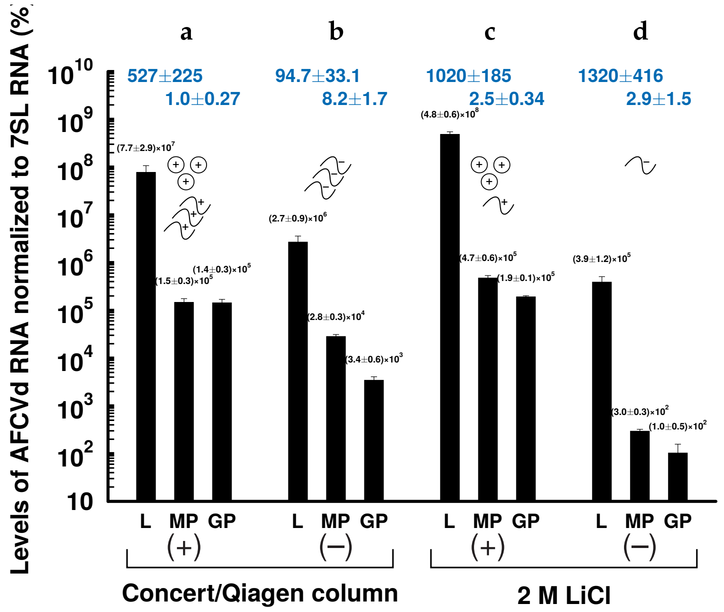

Figure S3. Comparative analysis of levels of AFCVd strands in leaves (L) versus mature (MP) and germinating pollen (GP) in plants transformed with pLAT52:AFCVd vector. Relative levels are given in percents after normalization to 7SL RNA. Reference level of (−) AFCVd strands in infected germinating tobacco pollen (the lowest level as in Figure 3) was taken as 100 %. Samples in a and b were purified by Concert reagent and through Qiagen columns, whereas samples in c and d were additionally concentrated from 2 M-LiCl soluble RNA (see Materials and Methods). Long replication intermediates and circular viroids were present in total RNA extracts (a and b), while replication intermediates are mostly non-soluble in 2 M LiCl (c and d); this feature is symbolized by the cartoons. Ratios of RNA levels are given in blue.

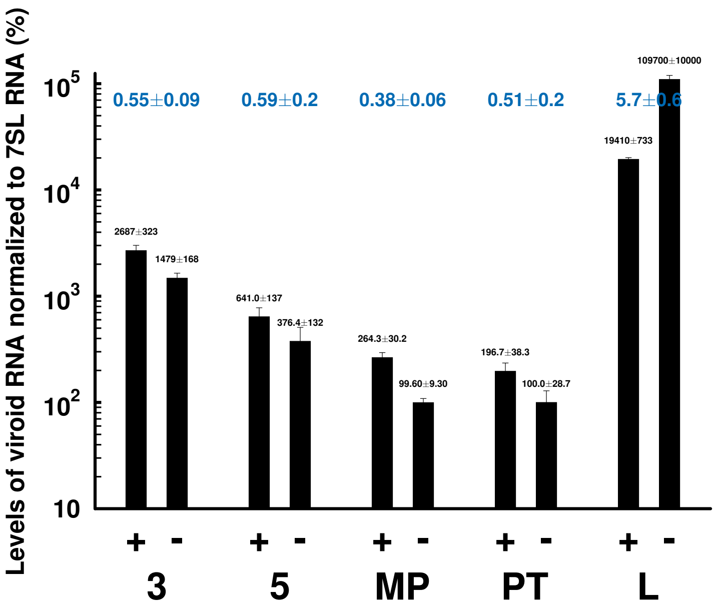

Figure S4. Comparative analysis of CBCVd (+) and (−) RNA levels in leaves (L) versus developing (stage 3 and 5), mature (MP) and germinating pollen (PT) in plants transformed with 35S:CBCVd vector. Relative levels are given in percents after normalization to 7SL RNA. Reference level of (−) CBCVd strands in transformed/infected germinating tobacco pollen (the lowest level) was set to 100 %. RNA was isolated by plant reagent and purified using Qiagen purification protocol and analyzed by strand-specific RT-qPCR (see Material and Methods). Ratios (−) to (+) chains are given for individual tissues in blue.

Table S3. Overview of all smallRNA sequence data sets before and after the preprocessing. Raw reads represent the amount of all sequenced reads; cleaned reads represent the amount of remaining reads after the trimming step by TRIMMOMATIC [9]. The percentages refer to the amount of raw reads. Sample abbreviations are as follows: L, K, V, leaves, control/healthy, AFCVd-infected, respectively; 3, 5, pollen, stages 3 and 5, respectively; Z, LAT52:AFCVd-transformed/infected; TG, pollen tubes; -1, -2, biological replicates.

sample	raw reads	clean reads	
L	11,287,184	6,541,037	(58 %)
K3-1	9,453,310	7,371,330	(78 %)
K3-2	11,507,521	8,794,233	(76 %)
V3-1	13,447,045	11,249,018	(84 %)
V3-2	9,725,947	6,231,046	(64 %)
K5-1	10,101,327	6,498,730	(64 %)
K5-2	9,751,223	7,545,789	(77 %)
V5-1	9,053,045	5,455,233	(60 %)
V5-2	15,718,151	8,584,379	(55 %)
ZL-1	19,316,194	7,364,597	(38 %)
ZL-2	12,557,978	5,532,267	(44 %)
ZTG-1	13,886,704	5,564,001	(40 %)
ZTG-2	13,211,578	8,368,058	(63 %)

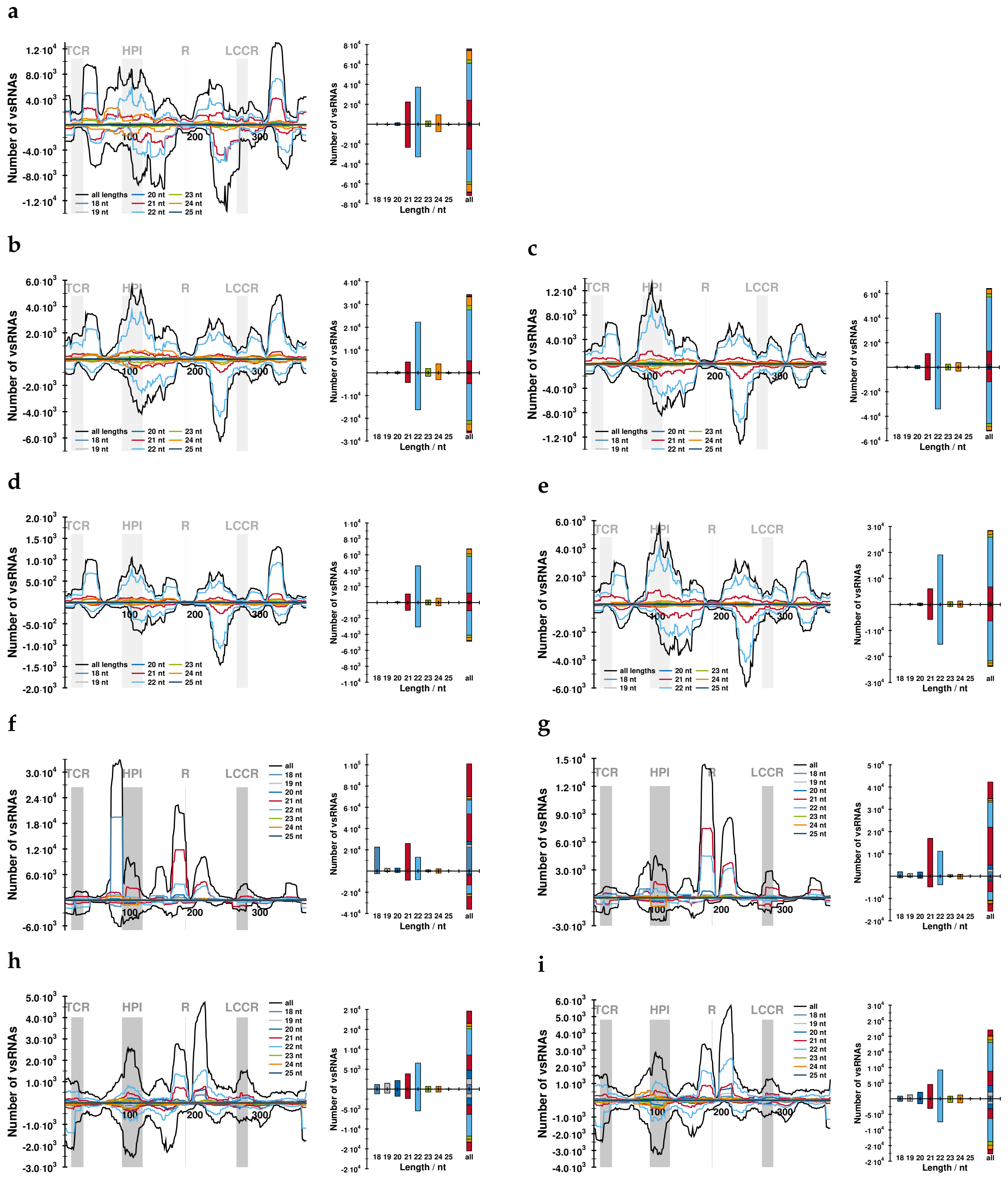

Figure S5. Mapping of vd-sRNA to the AFCVd genome and length distribution of vd-sRNA. Total RNA was isolated from infected leaves (a), infected pollen stages 3 (b,c) and 5 (d,e), and LAT52:AFCVd transgenic leaves (f,g) and LAT52:AFCVd transgenic pollen tubes (h,i) including biological replicates, purified and subjected to NGS; BOWTIE [10] allowing for one mismatch was used for mapping. For further details see Figure 6.

Table S4. Percentage of AFCVd mutations according to NGS. Total RNA was isolated from infected leaves (L), infected pollen stages 3 (V3-1, V3-2) and 5 (V5-1, V5-2), and LAT52:AFCVd transgenic leaves (ZL1, ZL2) and LAT52:AFCVd transgenic pollen tubes (ZTG1, ZTG2) including biological replicates, purified, subjected to NGS; bowtie allowing for one mismatch was used for mapping. Mutations are given if the ratio $\frac{\text{number of vd-sRNA with 1 error}}{\text{total number of vd-sRNA}} \cdot 100 > 10$. For further details see Figures 6 and S6.

Position	Mutation	L		V3-1		V3-2		V5-1		V5-2		ZL1		ZL2		ZTG1		ZTG2	
		(+)	(−)	(+)	(−)	(+)	(−)	(+)	(−)	(+)	(−)	(+)	(−)	(+)	(−)	(+)	(−)	(+)	(−)
67	G→U	80	76	32	34	69	61	41	49	62	46	13	32	22	40	0	0	0	0
77	G→A	10	9	62	41	35	18	39	13	40	18	85	17	12	3	3	2	8	0
196	U→C	12	6	7	3	7	1	0	0	4	1	24	0	33	0	21	3	24	1
207	U→A	4	22	39	30	24	20	48	39	51	45	78	50	76	45	70	52	71	61
306	C→A	12	65	9	20	30	47	26	46	26	57	6	1	8	3	0	0	0	0
	C→U	3	5	9	16	16	28	10	17	20	22	2	0	1	1	0	0	0	0
309	A→U	98	99	99	99	94	96	100	100	93	89	59	78	21	34	33	41	27	38
	A→C	0	0	0	0	6	3	0	0	7	10	36	21	65	64	65	53	68	55

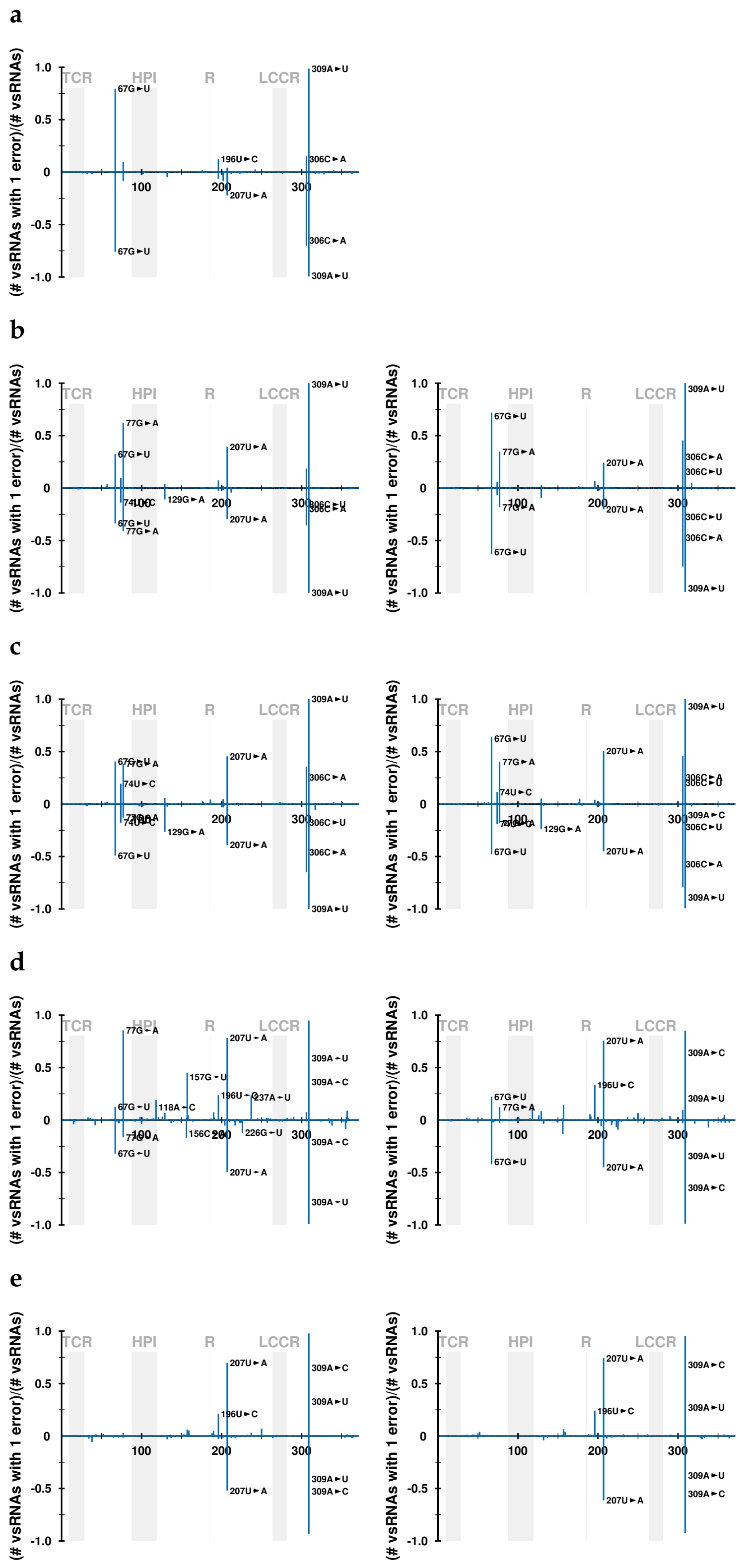

Figure S6. Position of mutations in the AFCVd genome according to vd-sRNAs. Total RNA was isolated from infected leaves (a), infected pollen stages 3 (b) and 5 (c), and LAT52:AFCVd transgenic leaves (d) and LAT52:AFCVd transgenic pollen tubes (e) including biological replicates, purified and subjected to NGS; bowtie allowing for one mismatch was used for mapping. Positions of mutations are labeled if the ratio $\frac{\text{number of vd-sRNA with 1 error}}{\text{total number of vd-sRNA}} > 0.1$. For further details see Figure 6.

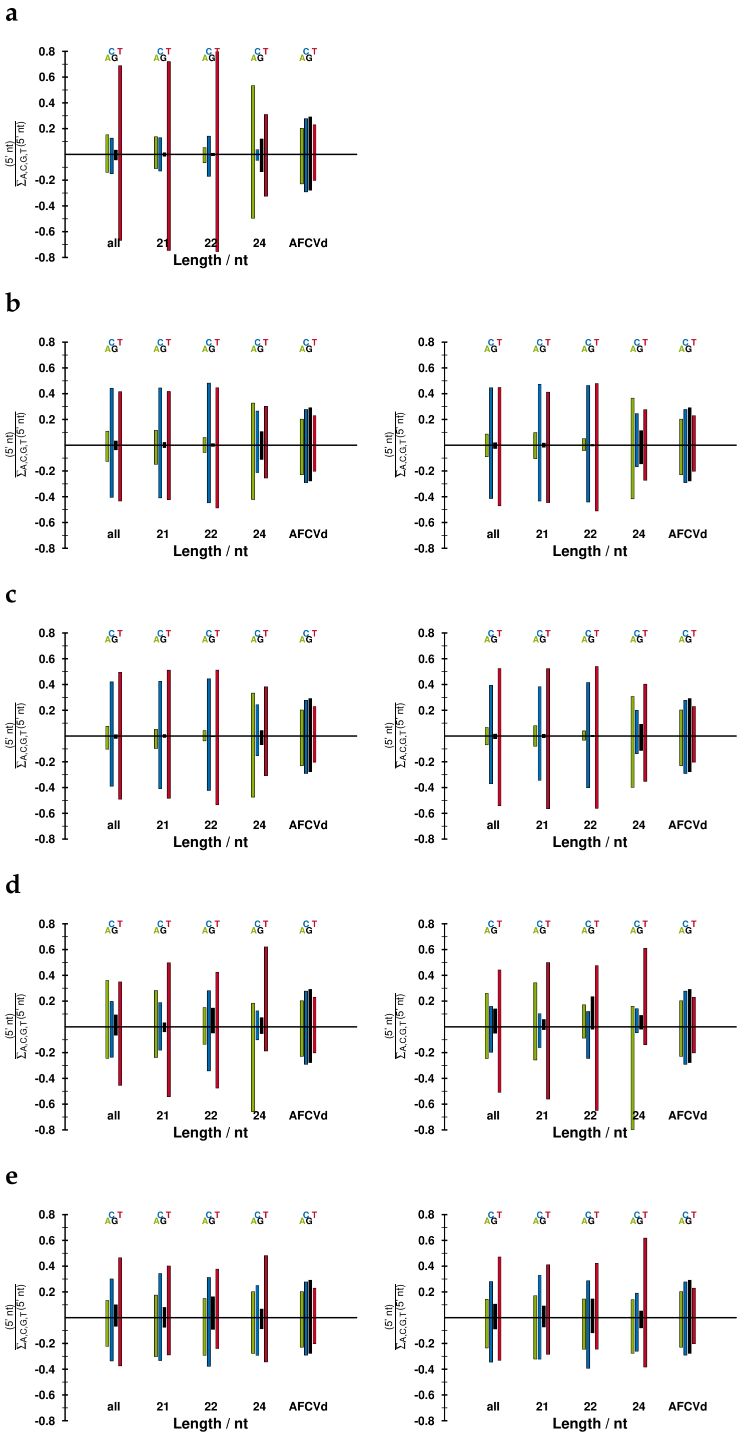

Figure S7. 5′ end nucleotide of vd-sRNAs. Total RNA was isolated from infected leaves (a), infected pollen stages 3 (b) and 5 (c), and LAT52:AFCVd transgenic leaves (d) and LAT52:AFCVd transgenic pollen tubes (e) including biological replicates, purified and subjected to NGS using bowtie allowing for one mismatch. For further details see Figure 6.

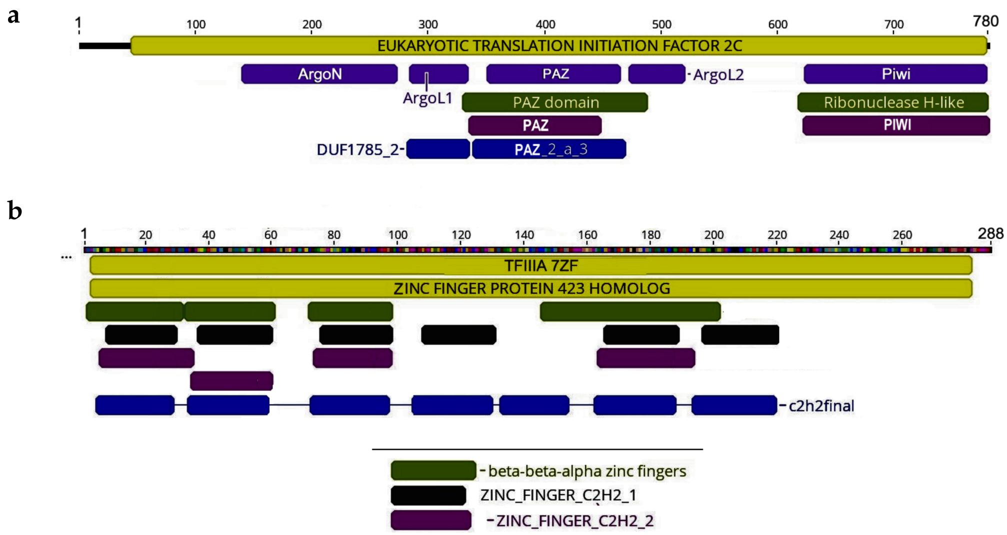

Figure S8. Protein domains of two factors cloned and analyzed in transient expression systems in this study. Domains were calculated and visualized using Geneious Prime 2020.04 software, Predict option using Inter Pro Scan. (a), *Nt*AGO5 from CBCVd transformed/infected tobacco pollen at developmental stage 3; (b), homolog of TFIIIA-7ZF isolated from *N. benthamiana* plants infected with lethal PSTVd strain AS1 [11].

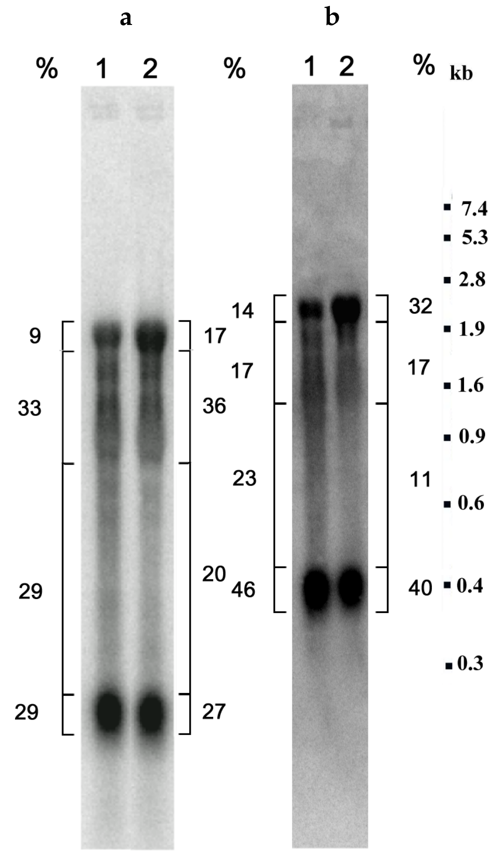

Figure S9. Spectrum and semiquantitative distribution of AFCVd (+)-genomic chains representing monomeric viroid and (+) replication intermediates in control-infected *N. benthamiana* leaf discs infiltrated with empty vector pJM14 (lane 1) or after co-expression of factor TFIIIA-7ZF (lane 2). Two independent experiments are shown in panels (a) and (b). Northern blot analysis was performed in 1.5 % agarose gels, 20 µg of RNA per sample were electrophoresed. After capillary transblotting to a Nylon Biodyne A membrane, nucleic were hybridized to strand-specific AFCVd(−) riboprobe to detect (+) AFCVd. Radioactivity signals were scanned and quantified using TYPHOON PhosphoImager (Amersham Biosciences, Sunnyvale, CA, USA) and quantified using ImageQuant software (Molecular Dynamics, Sunnyvale, CA, USA). The signal of individual gel zones, as indicated next to the gel slices, was calculated in percents. Positions of RNA marker in kb are shown on the right side of the panel.

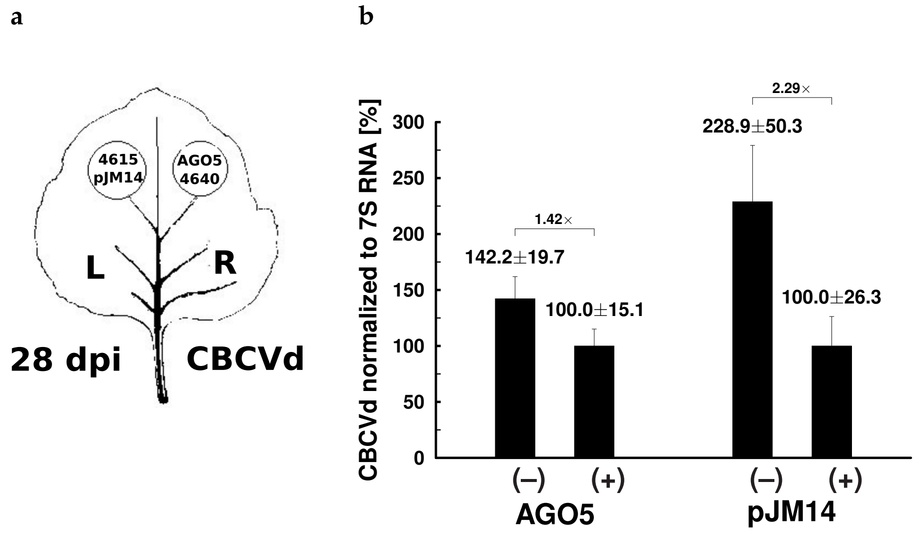

Figure S10. Levels of CBCVd (+) and CBCVd (−) RNA in leaf sectors of pre-infected *N. benthamiana* plants infiltrated in leaf halves either with control plant vector pJM14 or with *Nt*AGO5 cloned from developing viroid-infected pollen. After co-expression for 6 days, RNA was isolated using Concert plant reagent and purified via Qiagen columns (see Material and Methods). CBCVd was quantified using duplex strand-specific RT-qPCR (see Material and Methods). Results were normalized to 7SL RNA. (a) Scheme of infiltration; (b) results of quantification.

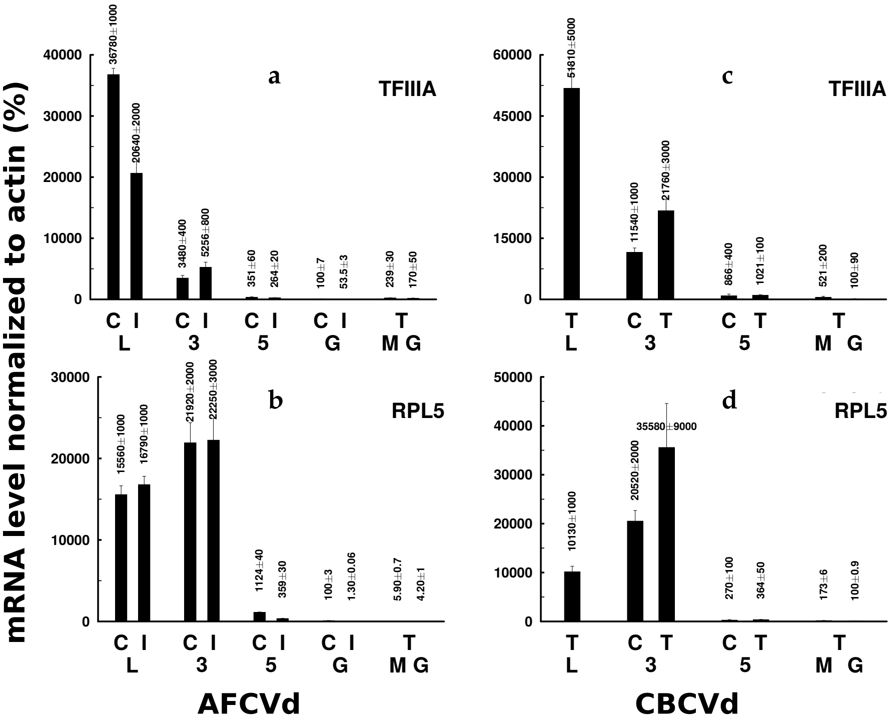

Figure S11. Relative mRNA levels of *Nt*TFIIIA (a, c) and *Nt*RPL5 (b, d) that could interfere with viroid replication cycle and/or viroid propagation in pollen. Data from qPCR, as described in Material and Methods, were normalized to actin.
(a), (b): samples from AFCVd-infected or pLAT58:AFCVd-transformed plants: C, healthy, uninfected, non-transformed controls; I, AFCVd-infected; T, transformed/infected; L, young leaves; 3, 5, pollen stages 3 and 5, respectively; G, mature germinating pollen; M, mature pollen.
(c), (d): samples from 35S:CBCVd-transformed/infected plants: C, healthy, uninfected, non-transformed controls; T, transformed/infected; L, young leaves; 3, 5, pollen stages 3 and 5, respectively; M, mature pollen; G, mature germinating pollen.

References

1. Wang, Y.; Qu, J.; Ji, S.; Wallace, A.; Wu, J.; Li, Y.; Gopalan, V.; Ding, B. A land plant-specific transcription factor directly enhances transcription of a pathogenic noncoding RNA template by DNA-dependent RNA polymerase II. *Plant Cell* **2016**, *28*, 1094–1107, [https://doi.org/10.1105/tpc.16.00100].
2. Matoušek, J.; Siglová, K.; Jakše, J.; Radišek, S.; Brass, J.; Tsushima, T.; Guček, T.; Duraisamy, G.; Sano, T.; Steger, G. Propagation and some physiological effects of *Citrus bark cracking viroid* and *Apple fruit crinkle viroid* in multiple infected hop (*Humulus lupulus* L.). *J. Plant Physiol.* **2017**, *213*, [https://doi.org/10.1016/j.jplph.2017.02.014].
3. Gibalová, A.; Steinbachová, L.; Hafidh, S.; Bláhová, V.; Gadiou, Z.; Michailidis, C.; Müller, K.; Pleskot, R.; Dupl'áková, N.; Honys, D. Characterization of pollen-expressed bZIP protein interactions and the role of ATbZIP18 in the male gametophyte. *Plant Reprod.* **2016**, *30*, 1–17, [https://doi.org/10.1007/s00497-016-0295-5].
4. Haseloff, J.; Siemering, K.; Prasher, D.; Hodge, S. Removal of a cryptic intron and subcellular localization of green fluorescent protein are required to mark transgenic *Arabidopsis* plants brightly. *Proc. Natl. Acad. Sci. U S A* **1997**, *94*, 2122–2127, [https://doi.org/10.1073/pnas.94.6.2122].
5. Matoušek, J.; Kocábek, T.; Patzak, J.; Füssy, Z.; Procházková, J.; Heyerick, A. Combinatorial analysis of lupulin gland transcription factors from R2R3Myb, bHLH and WDR families indicates a complex regulation of *chs*_H1 genes essential for prenylflavonoid biosynthesis in hop (*Humulus lupulus* L.). *BMC Plant Biol.* **2012**, *12*, 27, [https://doi.org/10.1186/1471-2229-12-27].
6. Matoušek, J.; Junker, V.; Vrba, L.; Schubert, J.; Patzak, J.; Steger, G. Molecular characterization and genome organization of 7SL RNA genes from hop (*Humulus lupulus* L.). *Gene* **1999**, *239*, 173–183, [https://doi.org/10.1016/S0378-1119(99)00352-2].
7. Matoušek, J.; Kocábek, T.; Patzak, J.; Bříza, J.; Siglová, K.; Mishra, A.; Duraisamy, G.; Týcová, A.; Ono, E.; Krofta, K. The "putative" role of transcription factors from *Hl*WRKY family in the regulation of the final steps of prenylflavonid and bitter acids biosynthesis in hop (*Humulus lupulus* L.). *Plant Mol. Biol.* **2016**, *92*, 263–277, [https://doi.org/10.1007/s11103-016-0510-7].
8. Baulcombe, D.; Molnár, A. Crystal structure of p19—a universal suppressor of RNA silencing. *Trends Biochem. Sci.* **2004**, *29*, 279–281, [https://doi.org/10.1016/j.tibs.2004.04.007].
9. Bolger, A.; Lohse, M.; Usadel, B. Trimmomatic: a flexible trimmer for Illumina sequence data. *Bioinformatics* **2014**, *30*, 2114–20, [https://doi.org/10.1093/bioinformatics/btu170].
10. Langmead, B.; Trapnell, C.; Pop, M.; Salzberg, S. Ultrafast and memory-efficient alignment of short DNA sequences to the human genome. *Genome Biol.* **2009**, *10*, R25, [https://doi.org/10.1186/gb-2009-10-3-r25].
11. Matoušek, J.; Kozlová, P.; Orctová, L.; Schmitz, A.; Pešina, K.; Bannach, O.; Diermann, D.; Steger, G.; Riesner, D. Accumulation of viroid-specific small RNAs and increase of nucleolytic activities linked to viroid-caused pathogenesis. *Biol. Chem.* **2007**, *388*, 1–13, [https://doi.org/10.1515/BC.2007.001].
